# Coupling causality and interpretable machine learning to reveal the reaction coordinate of C–N coupling with a supramolecular Cu-calix[8]arene catalyst

**DOI:** 10.1039/d5dd00216h

**Published:** 2025-09-02

**Authors:** R. A. Talmazan, J. Gamper, I. Castillo, T. S. Hofer, M. Podewitz

**Affiliations:** a Institute of Materials Chemistry, TU Wien Getreidemarkt 9 A-1060 Wien Austria maren.podewitz@tuwien.ac.at; b Institute of General, Inorganic and Theoretical Chemistry, Leopold Franzens University of Innsbruck Innrain 80/82 6020 Innsbruck Austria t.hofer@uibk.ac.at; c Instituto de Química, Universidad Nacional Autónoma de México, Ciudad Universitaria Ciudad de México 04510 Mexico

## Abstract

Supramolecular 3d transition-metal catalysts are large, flexible systems with intricate interactions, resulting in complex reaction coordinates. To capture their dynamic nature, we developed a broadly applicable, high-throughput workflow, that leverages quantum mechanics/molecular mechanics molecular dynamics (QM/MM MD) in explicit solvent, to investigate a Cu(i)-calix[8]arene-catalysed C–N coupling reaction. The system complexity and high amount of data generated from sampling the reaction requires automated analyses. To identify and quantify the reaction coordinate from noisy simulation trajectories, we applied interpretable machine learning techniques (Lasso, Random Forest, Logistic Regression) in a consensus model, alongside dimensionality reduction methods (PCA, LDA, tICA). By employing a Granger Causality model, we move beyond the traditional view of a reaction coordinate, by defining it instead as a sequence of molecular motions leading up to the reaction.

## Abstract 2

Interpretable machine learning coupled with causality analysis revealed the reaction coordinate of the C–N coupling step of a supramolecular Cu-calix[8]arene catalyst from explicit solvent QM/MM MD reaction trajectories.

## Graphical abstract

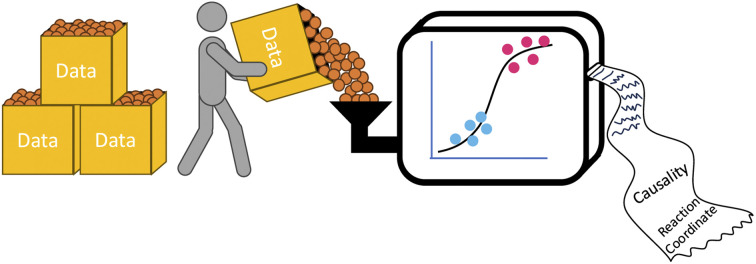

## Introduction

Nature has perfected the principle of catalysis in enzymes, where a precise control of the environment surrounding the catalytic centre and substrate lowers the energy barrier.^1^ In an effort to mimic the tight control over the environment, the field of supramolecular catalysis chemistry has emerged.^2–6^ For example, the use of a macrocycle, such as calix[*n*]arene, allows the substitution of precious metals with more abundant counterparts, while maintaining high catalytic performance.^7,8^

Complimentary to experimental advances, computational chemistry has played a key role in the design and understanding of catalytic systems, by elucidating reaction mechanisms.^9–12^ Despite considerable efforts, quantum chemistry is limited in its predictive abilities.^13^ As the systems grow in complexity, there is a need to improve the chemical model that describes the catalytic system in its environment.^14^ Often, the errors introduced by a too simplistic chemical model exceed those arising from the use of an approximate theoretical methodology such as Density Functional Theory (DFT).^13,15^ Consequently, the goal is to create a “digital twin” of the reaction flask that is an *in silico* model, which fully replicates experimental conditions: a catalyst in explicit solvent, at finite temperature and pressure. As a description with full *ab initio* quantum chemistry is not feasible, a tailored multiscale strategy is required, accounting for conformational flexibility, explicit solvation, and the dynamic nature of reactions.

Although many computational mechanistic studies are still performed on a single structure, conformer searches recently gained popularity^14,16,17^ thanks to easily accessible tools,^18,19^ facilitating a transition to structure ensembles, which provide a more complete picture of the reactivity.^14,17^ The standard implicit solvation model can favour unrealistic, very compact structures with many intramolecular hydrogen bonds.^7,19^ Ideally, a more realistic model would account for explicit solvent molecules either through a full condensed phase calculation or through a microsolvation approach.^20,21^

Molecular dynamics simulations can describe the dynamic nature of the catalysts bringing its description closer to *operando* conditions, which can reveal new insights into the reaction mechanism.^22–25^ While this treatment increases computational demands, by relying on multiscale methods, this hurdle can be greatly diminished. Quantum mechanics/molecular mechanics (QM/MM) models describe the catalytic centre and substrates at a QM level, while the surrounding environment is treated with MM.^26–28^ A transition to QM/MM molecular dynamics (MD) in explicit solvent allows for sampling timescales magnitudes higher than in a pure QM approach. Due to thermal fluctuations, repeated sampling is a requirement for statistically relevant information regarding energy barriers and structural information.

While the setup of such multiscale methods is a challenge in itself, a vast amount of data is generated from the simulations. Chemical knowledge can be extracted from these data, for example in the form of the reaction coordinate *i.e.*, the movements of atoms which take place as the chemical reaction proceeds. Knowledge of the reaction coordinate allows for further reduction in computational costs, by enabling the use of enhanced sampling methods geared towards overcoming large energy barriers, such as those observed in chemical reactions.^29^ The identification of the reaction coordinate from simulation data can be performed in a variety of ways.^30^ While machine learning (ML) approaches can be used to evaluate the data and extract condensed results from simulations, such as the committor function,^24,31^ complex neural network approaches generally are not directly interpretable.^32^ Whether through the use of convolutional neural networks or graph neural networks,^33,34^ a subsequent analysis is still needed to render the reaction coordinate interpretable. Instead, interpretable, or explainable, machine learning techniques, such as Decision Trees, Random Forests or Logistic Regression, as well as path sampling-based methods, such as predictive power analysis,^35,36^ offer good performance in extracting relevant information from large datasets and presenting them in easily understandable ways. In addition, dimensionality reduction techniques, such as Principal Component Analysis^37^ (PCA) or time-lagged Independent Component Analysis^38^ (tICA) effectively detect combined coordinates from the trajectories, revealing the key motions of a system. Furthermore, methods such as *k*-nearest neighbour and *t*-distributed stochastic neighbour embedding have shown some promise in dealing with complex datasets.^39–41^ Yet these dimensionality reduction techniques have almost exclusively been applied to biomolecules^42–44^ with few exceptions.^45,46^ A combination of aforementioned methods offers great promise to detect a cumulative reaction coordinate from a multitude of independent trajectories, providing chemical insight into the mechanism and reactivity of the system. However, to the best of our knowledge these combined methods have not been applied to study reaction mechanisms in explicit solvent, let alone large supramolecular transition-metal catalysts.

Another aspect that has until now been neglected in chemistry is causality. While the concept is widespread across various scientific domains^47^ – ranging from economics^48–52^ and climate research^53–57^ to biology^58,59^ and medical studies^60–62^ – it remains surprisingly absent in the field of chemistry. Although a handful of precedents in biomolecular simulations exists,^63–65^ it has not been explored to study chemical reactions, not to mention transition-metal catalysis. As MD simulation trajectories are essentially discrete time series, containing the various degrees of freedom of the system, causality can be statistically inferred from the analysis of these trajectories. Consequently, the reaction coordinate can be decomposed into a sequence of motions leading up to the reaction, exposing the intricate interplay of functional groups of the system, offering an unprecedented view of reactivity.

**Fig. 1:**
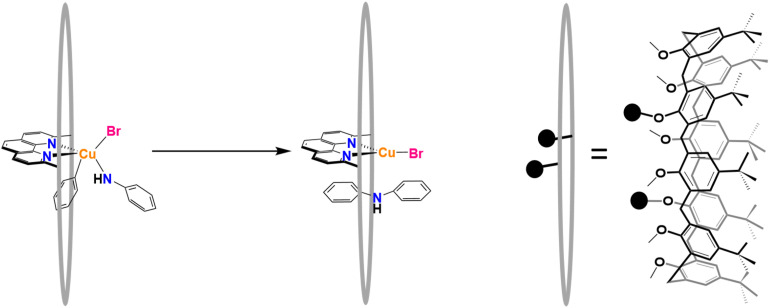
The supramolecular calix[8]arene-based [Cu(C_8_PhenMe_6_)I] system that catalyses the C–N coupling reaction of phenyl bromide and aniline. The black spheres represent the connections of the calixarene ring to the phenanthroline moiety.

A supramolecular catalyst that has shown remarkable catalytic efficacy for C–N coupling is the Cu(i)-1,5-(2,9-dimethyl-1,10-phenanthroyl)-2,3,4,6,7,8-hexamethyl-*p-tert*-butylcalix[8]arene, short noted as [Cu(C_8_PhenMe_6_)I].^7^ The macrocyclic ligand, sketched in Fig. 1, allows for usage of earth abundant metals, here Cu, which is an essential step towards more sustainable chemical processes.^66–71^ The investigated system necessitates explicit solvation for accurate results, as implicit solvation models lead to a collapse of the macrocyclic cage, which compromises catalytic activity (see also Fig. S14–S15, Table S5 in the SI).^7^ To account for the conformational flexibility of the supramolecular cage^7,72^ and the dynamic nature of the system as a whole, a dynamic, ensemble-based approach is required to study the reaction. While the mechanism of this catalyst was established to be a sequence of oxidative addition/reductive elimination,^7^ the dynamic effects of the system, in particular the contribution of the cage, are unknown. From previous studies, we know that explicit solvent molecules are crucial to maintain the shape and functionality of the cavity, however, we see no experimental or theoretical evidence of their participation in the reaction itself.^7,72^

We developed a multiscale QM/MM MD approach to understand the bond formation dynamics of the C–N coupling step with the Cu-calix[8]arene catalyst in explicit chloroform. By relying on the GFN2-xTB^73^ method to describe the QM part, we achieved massive sampling, resulting in 152 individual unbiased reaction trajectories. To extract chemically relevant information from these data, we employed supervised and unsupervised interpretable machine learning dimensionality reduction models, in order to identify the cumulative reaction coordinate and to detect critical movements in the structure. A consensus approach combining individual machine learning techniques improved the performance. The statistical Granger Causality analysis model^74,75^ was employed to decompose the reaction coordinate into a sequence of individual consecutive movements. Finally, Random Forest models and Decision Rules allowed us to quantify the reaction coordinate. This work serves as a broadly applicable template for any mechanistic investigation, revealing and quantifying complex reaction coordinates, along with causal effects derived from the individual movements leading up to the reaction.

## Results

We set-up a QM/MM model [Cu(C_8_PhenMe_6_)I] catalyst, where the reaction centre is modelled by QM and the macrocyclic ligand, as well as the solvent, by MM (see methods and SI Fig. S1 and Table S1 for details). We obtained 152 unbiased QM/MM MD reaction trajectories for the C–N coupling step O7 with a total of over 3 ns of simulation time. Out of these, 142 reacted spontaneously within 20 ps of simulation time without the need to bias. From these trajectories we evaluated the reaction energy and labelled the structural data accordingly as educt, transition state, or product – resulting in three ensembles. We then used this information to identify the reaction coordinate, identify a sequence of movements leading to the reaction, and quantify it.

### Reaction energetics analysis and ensemble labelling

We analysed 142 simulations, in which a reaction occurred, to gain insights about the C–N coupling process. As the reaction happened spontaneously during the simulations, the energy profile could be obtained directly (see SI Fig. S2) and was used to identify the three states, educt, transition state, and product. These states were labelled based on the energies, with the educts considerably higher in energy than the products. The transition states were labelled as the highest energy point, before the energy drop associated with C–N coupling occurred. For technical details see methods section and SI Section 2.

The reaction energy was obtained by averaging the ensembles of the educt and product states and calculating the difference; it amounts to −212 ± 25 kJ mol^−1^ (See SI Sections 3 for details regarding uncertainty estimation). A sigmoid fit through the smoothened energy profile of each simulation (see SI, Fig. S2B and S2C) allowed identification and the calculation of the energy barrier to be 13 ± 9 kJ mol^−1^. These GFN2-xTB reaction energies and structures are in excellent agreement with full DFT data, obtained with PBE0/def2-SVP/D3 (see SI Section 4, Fig. S3 and Table S2).

### Extracting chemical information from structural data

To obtain information about the changes in chemical structure from the reaction trajectories with a total of over 1.5 million frames, we resorted to interpretable machine learning approaches.

### Determination of a suitable coordinate system

A standard method to extract reaction coordinates from trajectories, either in biomolecular or reaction dynamics studies, is Principal Component Analysis (PCA)^37^ in cartesian coordinate space. However, this approach proved unsuccessful for the [Cu(C_8_PhenMe_6_)I] catalyst due to the difficulty in properly aligning this highly flexible system. The corresponding PCA does not show any separation between the three states (Fig. S4).

**Fig. 2:**
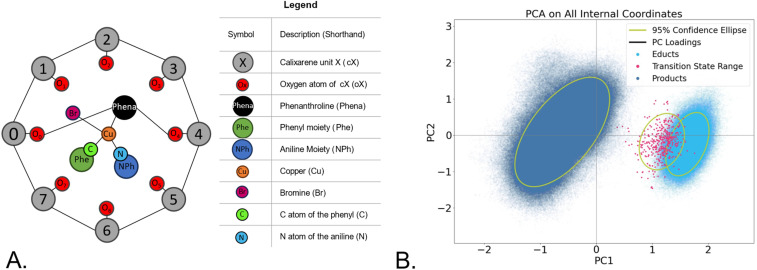
(A). Schematic depiction of the [Cu(C_8_PhenMe_6_)I] intermediate that we denoted as “educt” here, showing the centres of mass used for the calculation of the reduced set of internal coordinates; (B). PCA performed on the reduced internal coordinates.

To achieve good separation between the three states, educt, transition state, and product, we developed a reduced internal coordinate description of the system (see Fig. 2A) to minimize the noise from highly correlated coordinates.^76^ We used bond, angles, and torsion coordinates and described rigid fragments, such as the individual calixarene units (cX), the phenanthroline bridge (Phena), the phenyl (Phe) and aniline (NPh) moieties by their respective centres of mass (Fig. 2A). An overview over the distribution of the internal coordinates which define the reduced model can be found in the SI, Fig. S5. This internal coordinate set nicely separates educts and products in the PCA space (Fig. 2B), but still shows overlap between educts and transition states. Analysing the loadings of the principal components (see SI Tables S3 and S4), we can see that the main contributions belong to the coordinates defining the reaction centre (C–N, NPh–C, NPh–Phena distances), as well as to the distances between the product and the cage, describing changes in the coordination at the Cu centre as the product is formed and the settling of the product in the cavity.

### Improved reaction coordinate detection through supervised methods

**Fig. 3:**
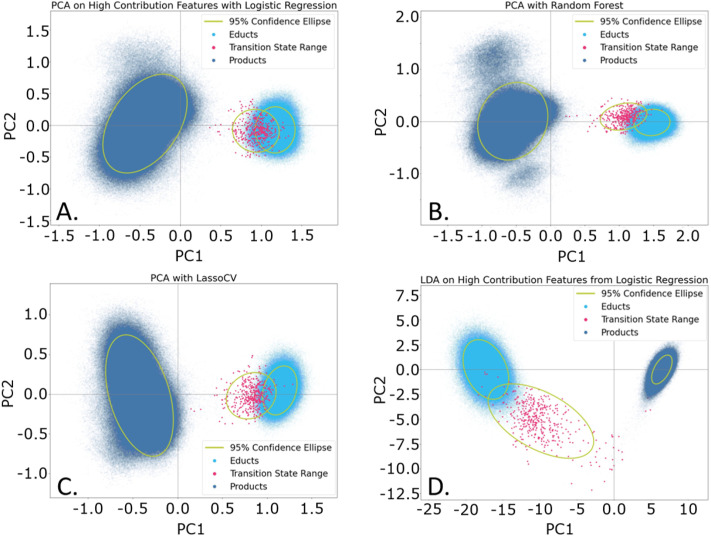
Dimensionality reduction performance with various feature reduction methods: (A). PCA with Logistic Regression with mean-based cutoff; (B). PCA with Random Forest with mean-based cutoff; (C). PCA with LassoCV; (D). LDA with Recursive Feature Elimination with cross validation using a Logistic Regression classifier.

We intended to further improve the separation of the three states in the PCA by utilizing the labelling of the data (See SI, Section 2), indicating each structure as educt, product or transition state. Using this information, we trained a model that maximized the separation between the three ensembles and simultaneously reduced the number of internal coordinates (features) to those that contribute the most to the separation. This process is known as feature elimination. There are several methods to achieve this, and we tested a few of them using PCA-based dimensionality reduction approaches (see Fig. 3). The results show that the performance of PCA varies depending on the feature elimination technique used (Fig. 3A–C and SI Fig. S6). However, good separation, even within the ensembles, can still be achieved, depending on the method of feature elimination applied.

A second method, Linear Discriminant Analysis (LDA),^77^ was also used for comparison. LDA is independent of feature selection and consistently yields excellent separation between groups (Fig. 3D and SI Fig. S6). While LDA produces compact, distinct ensembles, it does not provide separation within the ensembles themselves.

Analysis of the PCA loadings (see SI Table S3) revealed that amongst the top contributors to separating the three ensembles are the change in the distances defining the reaction centre (C–N, Phe–N, NPh–N, NPh-C and Phena-Cu-Br). Secondary features, such as the distance between the copper and O7, also plays an important role for the Logistic Regression (LR) classifier (Fig. 3A). The Random Forest (RF) identifies the C–N distance alongside Phe–N and NPh-C as important (Fig. 3B) with distinct product ensembles emerging. While in RF PC1 has the highest loading of all methods (0.68, see Table S3), the separation between the states is best for the LassoCV approach.

**Fig. 4:**
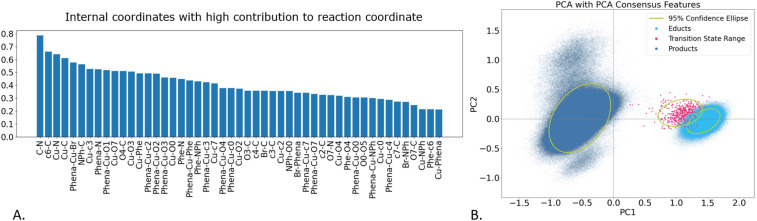
Analysis of internal coordinates deemed as highly important by the consensus model. (A) Internal coordinates and their contribution; (B) PCA of the consensus features.

As the feature selection methods differ in the selected internal coordinates and performance (see also SI Fig. S7 for a visual display of features across the methods), we switched to a consensus model (see methods for details on the creation of the consensus model). This consensus approach retains smaller number of features (49 internal coordinates), namely only those that were found to be of high importance in 75% of all previously used ML methods (Fig. 4A), that is in PCA and LDA models. For sake of comparison, the highly important features of a PCA-only and LDA-only consensus model are depicted in the SI in Fig. S8. It is important to note that individual models assign varying importance scores to each internal coordinate. As a result, the feature importance ranking derived from the overall consensus model is likely to differ from those of the individual models. We performed an additional PCA on the 49 consensus features (Fig. 4B). As evident from Table S3, in this model PC1 shows the highest retained variance (0.76) of all PCAs.

Judging the importance of the consensus model features (Fig. 4A), we see that the C–N bond distance is most important (in agreement with chemical intuition), alongside several distances and angles describing to the reaction centre. Notably, the c6–C distance is also deemed highly important, which indicates that the cage indeed plays a role in defining the reaction coordinate. This small set of internal coordinates yields almost perfect separation of the three states, as well as distinguishing the product conformations (Fig. 4B), thereby outperforming any of the individual feature selection methods.

### Time correlation-guided identification of slow system movements

While PCA focuses on the largest variance in the dataset, tICA can be used to separate and extract the internal coordinates which exhibit the strongest time-correlations for a chosen lag time, thus revealing slow movements in the system.

**Fig. 5:**
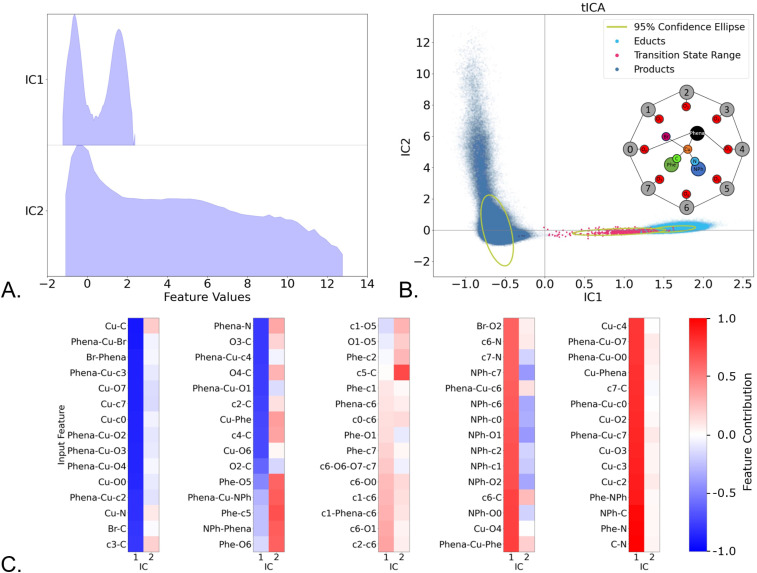
Time-lagged independent component analysis of the MD trajectories, using a lag time of 20 fs. (A) Distribution of the structural ensembles (feature values) over the first two independent components; (B). Projection of the ensembles in the space of independent components 1 and 2 (IC1 and IC2), with the ensemble colouring performed *a posteriori*; (C). Normalized internal coordinate (feature) contribution to each independent component.

Employing tICA on the initial reduced internal coordinate set (Fig. 2A), resulted in a good separation of the product and educt states, mainly across the first independent component (IC1), as shown in Fig. 5A, where the values of IC1 and IC2 are plotted separately, and Fig. 5B, where they are plotted against each other and the structures are color-coded according to their labels. Yet the transition state ensemble cannot be fully separated from the educts. When taking into consideration IC2 (Fig. 5A and B), we observe a broad distribution of the product ensemble, indicating significant conformational flexibility. The contributions to ICs can be traced down, by relating the contribution strength (Fig. 5C) to the degree of freedom it corresponds to (Fig. 5B insert). A positive contribution (coloured in red) means that the respective feature values increase as the values of the IC increases, while a negative contribution (coloured in blue) means the feature values decreases as the IC values increase. The absolute value of a contribution (colour intensity) represents the importance of the feature in defining the IC. Please note that going from the educt to the product corresponds to a decrease in IC1.

IC1 reveals the changes at the reaction centre related the Cu adopting a planar configuration upon product formation, as evident for example by the Phena-Cu-Br angle (1st column) that increases with decreasing IC1 (1st column). Of particular interest are the increase in Cu–C and the Cu–N bond distances (1st column) when going to the products, accompanied to a shortening of C–N (5th column), as indicative of the reductive elimination step, as well as the strong contributions of the distances between the aniline product moiety (NPh) and the c0, c2, c6 and c7 calixarene units (4th column). The later hint at the formation of π–π interactions between the cage and the product. Additionally, a tilting of the calixarene cage can be inferred, when looking at the changes in the Phena-Cu-cX and Phena-Cu-oX angles: For example, Phena-Cu-O2, Phena-Cu-O3, and Phena-Cu-O4 (1st column), all located at the lower rim of the calixarene cage (Fig. 5B (insert)), anticorrelate with IC1, hence, they increase when the product is formed (decreasing IC1), whereas those on the lower rim of the cage, such as Phena-Cu-c6 (4th column) as well as Phena-Cu-O7 and Phena-Cu-c7 (5th column) decrease. This finding indicates the movement in opposing directions, when looking at units on opposite sides of the cage. IC2 acts to separate various conformers within the product ensemble, where we can see a difference in the position of the product in the calixarene cage, inferred from the strength of the contributions of the product-calixarene unit distances. To further corroborate π–π interactions, we clustered the product ensemble and performed an NCIPlot analysis^78^ of the noncovalent interactions, which revealed weakly attracting van-der Waals interactions between the formed coupling product and the cage (see SI Fig. S16–S20).

### Revealing causality in the reaction coordinate

While the correlation analysis shows which movements take place in a correlated fashion, it is also interesting to evaluate the causality of these movements and how they propagate through the system. Given that tICA, a time-lag-based method, provided new insights into cage movement and product interaction, we applied the Granger Causality (GC) model, another time-lag-based approach, to assess the reaction trajectories for causal relationships. GC determines whether the prediction of a variable *X* in the future improves by including past events of *Y*. If this is the case, *Y* is found to Granger cause *X*.

With this causality model, we utilized the 49 consensus features (Fig. 4) and analysed each trajectory separately. Therefore, we can assess which each feature is influenced by which of the other 48 features; however, a complete analysis of all features quickly becomes impractical. Having identified C–N bond formation and changes in Cu-coordination as the most critical factors during product formation, we focused our GC analysis on the C–N feature. We analysed which of the remaining 48 features influenced the C–N feature and evaluated how often each of the 48 variables was found to Granger-cause C–N across all trajectories. For an overview over the 49 × 49 causality matrices generated for each reaction trajectory, the reader is referred to the GitHub.

**Fig. 6:**
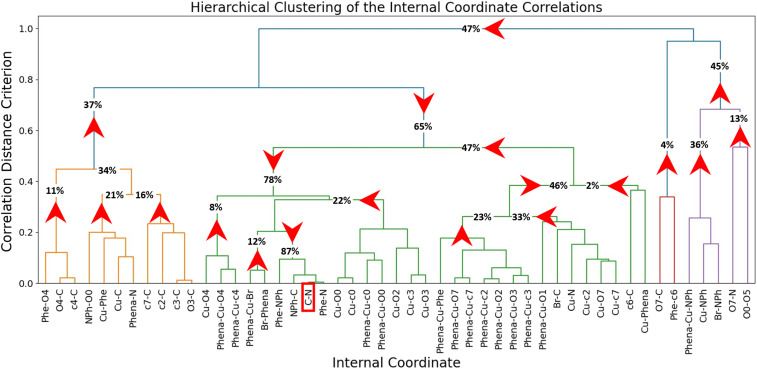
Propagation cluster map of the system, leading to the C–N bond formation. The numbers represent the total causality effects uncovered by the respective coordinate(s) and the red arrows indicate the direction of causality.

In addition, we performed a hierarchical clustering of the consensus features (branches in Fig. 6), which tells us, what features are correlated. Further insight can be obtained by quantifying the correlation with a Pearson score, resulting in a clustermap (Fig. S9 SI), which also reorders the internal coordinates according to their correlation to each other. From this we can observe two distinct regions in the catalyst, corresponding largely to the upper and lower rim of the calixarene cage.

Combing the information of the correlation with Granger Causality, we analysed the causality for C–N coupling, *i.e.*, C–N bond shortening, not only for the individual features but also for the combined branches (Fig. 6). While the red arrows in Fig. 6 show the direction of causality the percentages indicate in how many trajectories C–N bond formation was caused by the respective feature or group of features. Fig. 6 can be read as a map of movements leading to the coupling reaction, by choosing a starting point and following the arrows towards the highlighted C–N feature.

For example, in 11% of all trajectories C–N coupling is caused by a change in Phe-O4, O4-C, and c4-C distances (most left part of orange cluster, Fig. 6). Combining all features in the orange cluster, in 37% of all trajectories, the coupling is triggered by a move of features summarized in this cluster, consisting of the calixarene 2,3,4 and 7 to C distances as well as Cu-Phe distance. When we include information about the Phena-Cu-Br angle, alongside Cu and Br distances to NPh, from the purple cluster (right hand side of Fig. 6), as well as information from the red cluster, we can infer causality in 65% of all trajectories. The green cluster consists mostly of information related to the position of the calixarenes around the reaction centre. We see that changes in the Phena-Cu-calixarene angles (*e.g.*, Phena-Cu-O7, Phena-Cu-O2, …) on the upper and lower rim are correlated. Combined with the information from the clustermap (Fig. S9), we see that the angle with the upper rim decreases (positive Pearson correlation with C–N coupling, coloured in red), whereas those on the lower rim increases (negative Pearson correlation, coloured in blue), indicative of a tilt of the calixarene cage. When we combine all information together, we can infer that the C–N coupling is caused by movements in the calixarene cage, alongside the Cu coordination change in 87% of the trajectories. Please note that because of the correlation of the individual features, percentages of the combined features do not necessarily equal the sum of the individual contribution.

In addition to C–N coupling, the Phena-Cu-Br angle was selected as a high importance feature by the consensus model (Fig. 4A), it's the 5th in the ranking and the first angle, and we subjected it to GC analysis. It also corresponds to an intuitive view of the change in coordination around the copper. From the correlation analysis and clustering, it relates closely to the C–N bond shortening and it directly Granger-causes C–N coupling 12% of the time. Conversely, we found that the C–N distance shortening does not cause the change in the Phena-Cu-Br angles, allowing us to deduce that the two movements happen either simultaneously or, more likely, the change in the angle precedes the C–N bond shortening. Furthermore, we observed that movements of the calixarene cage Granger-cause changes in the Phena-Cu-Br angle in 72% of all trajectories, establishing the sequence of cage movement – Phena-Cu-Br change – C–N-shortening. Due to the increasing complexity, we opted not to conduct further analysis of the GC matrices.

### Quantification of reaction coordinates

While the consensus approach revealed the relevant internal degrees of freedom that define the reaction coordinate, as a next step, we sought to quantify it, by identifying ranges of individual features that separate the data into three ensembles. To achieve this, we used Decision Trees, which split ensembles by applying cut-offs to those coordinates that show the largest distribution differences between classes.

**Fig. 7:**
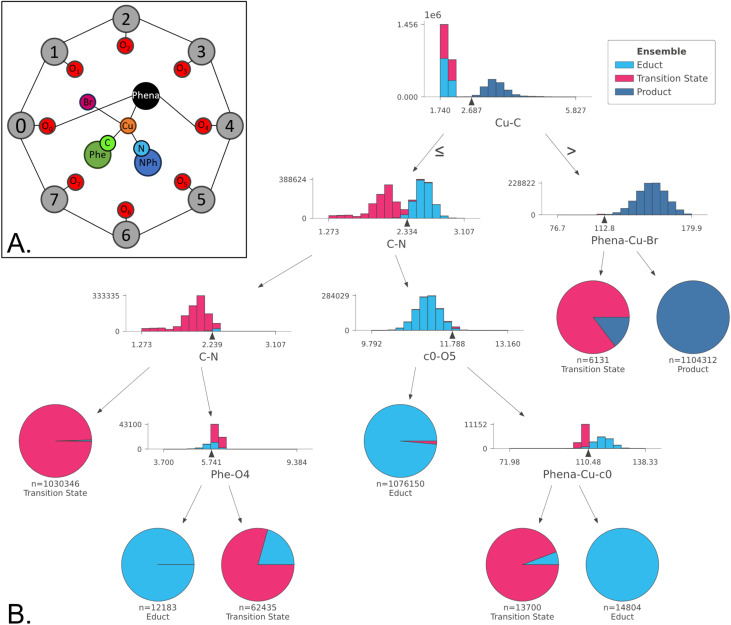
(A). Model of the Cu-calix[8]arene catalyst with the reduced centres of mass; (B). Decision Tree classifier used to interpret the differences between the 3 structure ensembles, trained on the balanced classes. As indicated by the inequality operators, structures with values smaller or equal to the threshold are summarized in the left branch, those with larger values on in the right branch.

The Decision Tree in Fig. 7 has been trained on the whole dataset. To avoid biasing against the transition state ensemble, which contains significantly fewer structures, balanced weight is given to all classes *via* oversampling. For comparison, the results of an unbalanced tree can be found in Fig. S10.

Analysing the tree, we can see that the Cu–C distance plays a key role in the splitting of the educts and products, with the majority of transition states being grouped with the educt class, indicative of an early transition state. The remaining transition state contamination of the product ensemble can be separated by taking the angle determined by the phenanthroline bridge, Cu and Br atoms into account (Phena-Cu-Br), where values below 112.8° are indicative of a transition state.

To differentiate between the transition states and educts, the C–N bond represents an effective metric, where values higher than 2.33 Å indicate an educt, while distances below indicate a transition state. The educt states that do exhibit a C–N bond distance similar to that of the transition state can be identified by a smaller phenyl to calixarene unit 4 oxygen atom distance (Phe–O4). When transition states exhibit a C–N bond distance over 2.33 Å, they can be distinguished from the educts by the calixarene c0 and calixarene O5 distance greater than 11.79 Å and the angle defined by the phenanthroline bridge, Cu and c0 (Phena-Cu-c0) smaller than 110.5°.

**Fig. 8:**
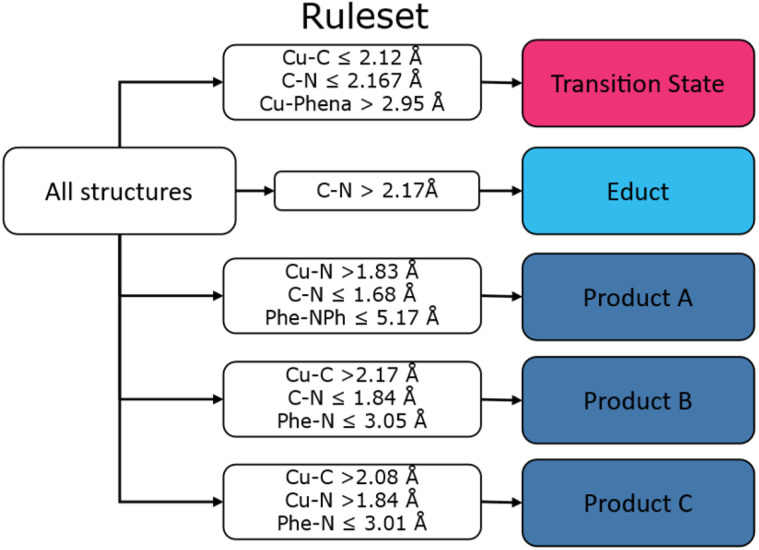
Graphical depiction of the Decision Rules derived from the RF approach, Product A, B, C refers to different conformer ensembles within the product category.

A major shortcoming of Decision Trees is that their results depend on the initiation conditions. However, their reliability can be improved by utilizing many Decision Trees in a Random Forest (RF) classifier and averaging the results. In general, this improves accuracy, but reduces the interpretability. To overcome this limitation Decision Rules can be deduced from the results, providing a semantic understanding of the RF classifier. We used 30 Decision Trees, each trained on a subset of the data, to yield the RF. When applied to our dataset, this method provides rules for each of the three classes, as seen in Fig. 8, below. A complete diagram of the Decision Rules can be found in the SI, Fig. S11.

Notably, the Decision Rules approach identifies three distinct rule sets for defining a product. Color-coding these three product states in the PCA with consensus showed clear separation (see SI, Fig. S12), which was further confirmed by kmeans clustering, where only minor overlap occurred. Hence, these three states are distinguishable to some degree.

## Discussion

Using semi-empirical quantum chemistry methods, we generated massive sampling of the reductive elimination step of the C–N coupling reaction with the [Cu(C_8_PhenMe_6_)I] catalyst, through a hybrid QM/MM molecular dynamics approach. Since the amount of generated data, alongside the very high dimensionality, makes the reaction difficult to interpret by visual analysis, statistical methods and machine learning techniques were used to extract chemically relevant information from the dataset.

As we observed a convergence of the PCA with increased sampling (see SI, Fig. S13), we assumed the total simulation time to be sufficient. In addition, we observed spontaneous C–N coupling in 142 of the 152 trajectories, hence, we could directly analyse these unbiased simulations.

By analysing the reaction energy profiles, we were able to quantify the reaction energy, as well as the reaction barrier, including uncertainty values. The computed reaction barrier amounted to 13 ± 9 kJ mol^−1^, which is within 6 kJ mol^−1^ of that obtained from the full DFT trajectories (18 kJ mol^−1^). Notably, the static quantum chemical (DFT) approach yielded a slightly higher barrier of 23 kJ mol^−1^,^7^ indicating that dynamics lower the barrier. For the reaction energy, differences are more pronounced with −212 ± 25 kJ mol^−1^ obtained with QM/MM MD *vs.* −255 kJ mol^−1^ with static DFT.^7^ However, these differences are due to the different conditions modelled: In the static DFT model, the studied structure was obtained at 0 K, which represents the bottom of the potential energy surface, while in this QM/MM MD study not only the average over all conformations is taken into account, but also the thermal energy, so the structures are not 0 K structures and therefore no minima on the potential energy surface. In addition, we assume that the product can undergo slow relaxation to lower energy conformations, which are too slow to observe in the timescales investigated here and which further contribute to the difference in reaction energies decreasing the variance between the static and the dynamic approach. Besides energetics, the reaction profile allowed for the categorization of the structures into three ensembles, namely educt, transition state, and product. This step was a key in improving the reaction coordinate recognition, as it allowed for the use of supervised learning methods to reduce the coordinate space. Due to the large energy difference between reactants and products, which renders the transformation irreversible in the context of unbiased MD at 300 K, we sampled a single chemical reaction per trajectory. Thus, there is one transition state structure before the large drop in energy and, as we have not biased our simulation, this structure should be on the dividing surface. Naturally, structures very similar to the transition state are likely to be found in the sampling leading up to the identified transition state due to recrossing events. Such transition state and transition state-like structures could probably be identified using a structural criterion and, in an iterative process, be removed from the educt ensemble. However, in the present case, we observe a good separation between transition state and reactant ensembles, also visible in the analysis of the internal coordinate distribution (SI Fig. S5), where for example, the C–N or the Phe–N coordinate show excellent separation of the three states. This finding indicates that there is no large transition state structure contamination within the reactant ensemble.

While PCA has been used in the past to determine the reaction coordinates, we have demonstrated that it is insufficient for a highly complex system, with many degrees of freedom. To yield any separation between the three states in the PCA, we had to transform cartesian coordinates to a set of internal coordinates, which we further reduced to minimize the number of highly correlated coordinates in the data set, thus reducing noise. Although this single step of our workflow is not fully automated at the moment, clear guidelines to obtain the reduced coordinate set can be applied, (i) usage of internal coordinates and (ii) representation of rigid groups such as phenyl by their centre of mass, which are applicable to describe any chemical system. While product states could be separated from educts and transition states, the latter two still showed overlap. In contrast, standard PCA on the cartesian coordinates resulted in no separation of the three states. We suspect the poor performance stems from failure to fully eliminate rotational and translational degrees of freedom from the system.

Utilizing the labelled data in PCA and LDA combined with supervised ML approaches resulted in a much better separation of the three ensembles. While the performance of PCA was highly dependent on the internal coordinate set, LDA showed remarkable separation between the three ensembles, highlighting the robustness of the method. A consensus model developed to combine the performance of the various dimensionality and feature reduction methods, identified 49 internal coordinates to be relevant, with the C–N distance being the most prominent one, which is in agreement with chemical intuition.

From looking at the cumulative results of all analyses performed on the reaction trajectories we deduced which movements took place during the reaction. From the PCA, LDA and the correlation analysis (clustermap), we saw a tilt in the calixarene cage. Likewise, we observed changes in the coordination around the Cu centre, as indicated for example by Phena-Cu-Br angle changes and the C–N bond shortening upon product formation.

**Fig. 9:**
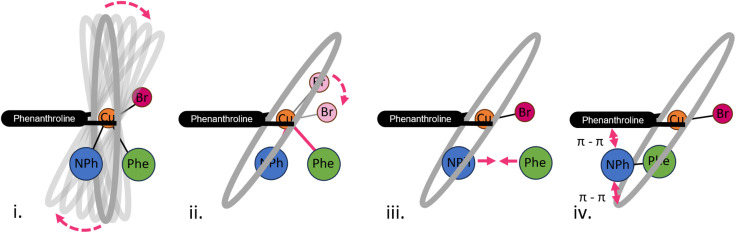
Schematic representation of the movements corresponding to the reaction coordinate of the C–N coupling reaction. (i) Tilting movement of the calixarene cage; (ii) Change in coordination of the copper centre; (iii) Shortening of the C–N bond; (iv) π–π stacking effects stabilise the product in the cavity.

Complementary to PCA, tICA was used to identify slow movements of the system. While tICA provided structural insights into the product conformer ensemble, it failed to distinguish educts from transition states. Thus, tICA was insufficient fully identify the reaction coordinate Finally, the tICA results suggest a settling of the product in the calixarene cage, governed by π–π stacking interactions, which was further corroborated by analysis of non-covalent interactions of representative structures (SI Section 12 Fig. S16–S20). These correlations can be then temporally defined by interpreting the Granger causality analysis. Through the causality analysis of changes in the C–N bond, we deduce that the reaction is Granger-caused by a movement in the reaction centre (such as a change in the Phena-Cu-Br angle) or a change in the calixarene cavity conformation, particularly calixarene units 2,3 and 4, whereas in turn changes in the Phena-Cu-Br angle are Granger-caused by changes in the calixarene conformation. Finally, the π–π stacking increased once the formed product reoriented itself inside the cavity. Therefore, we may create a sequential image of the reaction, schematically depicted in Fig. 9 as follows: (i). The calixarene cage tilts perpendicularly to the phenanthroline; (ii). The change in Cu-coordination takes place before the C–N distance shortening, but after the tilt of the cage; (iii). The C–N bond distance shortens; (iv). π–π stacking effects drive the movement of the product inside the cage and below the phenanthroline.

To the best of our knowledge, this study represents the first application of Granger Causality analysis to a complex chemical reaction – specifically, the C–N coupling reaction step catalysed by [Cu(C_8_PhenMe_6_)I] – and serves as a proof of principle. However, the model provides a simple interpretation of causality, as it analyses pairs of variables and not more complex composite variables. Transitioning to more advanced causal discovery methods, such as PC Momentary Conditional Independence (PCMCI)-based approaches,^79^ would enable better interpretation of nonlinearity and the presence of hidden variables within the dataset. Nonetheless, such advancements are beyond the scope of this work.

To quantify the changes in internal coordinates, we employed a Decision Tree trained on the three ensembles, identifying the Cu–C and C–N distances as main feature for separating products from educts and transition states, respectively. While Decision Trees are sensitive on the initialisation condition and require size limitations to maintain interpretability, a Random Forest (RF) approach mitigates these limitations by aggregating multiple trees. Applying the Decision Rule method then allows for the semantic interpretation to a RF classifier, effectively separating the three ensembles. In addition, three distinct product conformers are identified, each defined by a unique set of decision rules and distinguishable in the PCA consensus features plot (Fig. S12) as well as in IC2 of the tICA analysis. Structural differences are related to changes in cage conformations. These conformations will likely converge as the product diffuses out of the cavity.

To the best of our knowledge there is only a single study of the reaction dynamics of a transition-metal complex with explicit solvent with repeated sampling of the reaction step.^24^ This study on Fe-oxo-mediated C–H functionalization reactions by Joy *et al.* used kinetic energies, quantum numbers and velocities to distinguish between two different dynamic reaction pathways.^24^ While they also used ML for feature selection, their focus is on physical chemical factors that impact reactivity. Our focus lies on the investigation of structural changes to ease interpretation and to facilitate causality analysis, which has never been attempted for chemical reactions, but opens a completely new angle on how to understand reactivity. From all statistical analyses, we can see that the transition state structures tend to be close to the educt or even overlay with the educt space: for example, this is visible in the unsupervised PCA (Fig. 2), supervised PCA (Fig. 3) or tICA (Fig. 5), as well as in the histograms of all internal coordinates (SI Fig. S5), where educt and transition state distributions overlap for many internal coordinates, while being clearly separated from the product. We surmise that this is an indication that we have an early transition state here – information that can be utilised in further optimisation of the catalyst. While the reaction step under investigation does not represent the rate-determining step, as previously reported, the energy for release of the product from the catalyst is notably reduced by the presence of the calixarene cage, allowing for improved catalytic performance.^7^ Our analysis revealed that the reaction starts with a tilt in the cage. Therefore, modification of this moiety distal to the reaction centre can affect reactivity and even selectivity, similar to the allosteric effects seen in natural enzymes. Experimentally, modifications could include changing the size and rigidity of the cage, or introducing asymmetric modifications to one side of the cage. All of these efforts can result in greater control over substrate selectivity. Identifying possible allosteric sites in other systems could help advance supramolecular chemistry in rational design, which is of particular interest for homogeneous catalysis, where such insights may lead to greater substrate selectivity or more efficient catalysts.

Our study presents a proof-of-concept approach to the discovery of complex reaction coordinates that is transferable to any chemical reaction. The presented methodology is not only applicable to the study of single reaction steps, but can be extended to reactions with competing reaction pathways that determine selectivity: If the reaction energy profile is different, then the energy can be used as a simple criterion to group the trajectories and separate the pathways. In case, the energy differences are similar and only the product structures are different, this will be highlighted by PCA or tICA, allowing separation using a clustering approach. This information can then be leveraged to either separate the sampling data and categorize the reaction trajectories or to perform new, biased or restrained simulations which direct the reaction mechanism through a single pathway.

Our protocol can be integrated into computational chemistry workflows to investigate the reactivity of complex systems under *operando* conditions. By identifying secondary contributions to the reaction coordinate, such as remote conformational changes, it can guide further studies and inform the design of reactivities and selectivity by tracing the impact of modifications on the reaction profile under *operando* conditions. Consequently, additional insights can be obtained from reaction trajectories with minimal additional computational effort.

## Conclusion

We developed a workflow to identify and quantify the reaction coordinate from a set of trajectories, detecting chemically intuitive and remote contributions to the reactivity.

By devising a high throughput QM/MM MD workflow, we were able to study the C–N coupling reaction dynamics of a supramolecular Cu-calix[8]arene catalyst under experimental conditions. This development is a crucial step towards a predictive *operando* model for complex catalytic reactions. It allowed us to extract not only reaction energies and barriers with uncertainties, but also provides insights into the intricate dynamic nature of the macrocyclic transition-metal catalyst in explicit solvent.

Interpretable machine learning techniques have proven invaluable in dealing with the vast amount of data, because of their ability to trace results back to structural changes. However, a consensus model is needed to identify the internal coordinates with the highest contribution to separating educt, transition state and product, and to eliminate the inherent variability and instability of individual ML approaches.

By performing a causality analysis of the internal coordinates of the consensus model, an extra temporal dimension can be added to the reaction coordinate, allowing us to explain the chemical reaction as a sequence of movements leading up to C–N bond formation. This information, pinpointing the exact source (group of atoms) that triggers the reaction, allows the experimental chemist to generate testable hypotheses to enhance reactivity, for example by suitable chemical modifications. By checking the outcome of Decision Trees and Decision Rules run on this modified system, we can gauge the impact of a specific change on the reaction coordinate. The methodology is implemented in a python script and presented in the Jupyter notebook provided on github (see Data availability Section). The analysis is largely automated, requiring only a few inputs from the user, namely: (i) system trajectories; (ii) coordinates which should be considered for dimensionality reduction *e.g.* centres of mass for rings; (iii) labelling criteria for the various states of the system; (iv) cutoff values for the consensus model. Notably, the labelling of the states can be done on any descriptor of the system, including energetic criteria, as well as partial charges or other molecular properties. As a result, it can be readily applied to other systems as a digital tool. By limiting the analysis to structural changes in the system, we believe this technique is accessible to non-expert users. The results reflect coordinate changes during the reaction, which makes them easy to understand for a general chemist.

Our methodology was demonstrated on a highly flexible Cu-calix[8]arene catalyst. However, the approach supports the exploration of both covalent and non-covalent interactions, allowing for the applicability to the investigation of other phenomena such as cluster formation and aggregation. Nevertheless, a requirement is that structures can be labelled, using either energetic criteria or any molecular feature. If that is given, it offers a broadly applicable framework for probing reaction coordinates in a variety of dynamic chemical systems, ranging from small (in)organic complexes to more intricate biomolecular structures.

## Methods

### Workflow for determination of complex reaction coordinates

**Fig. 10:**
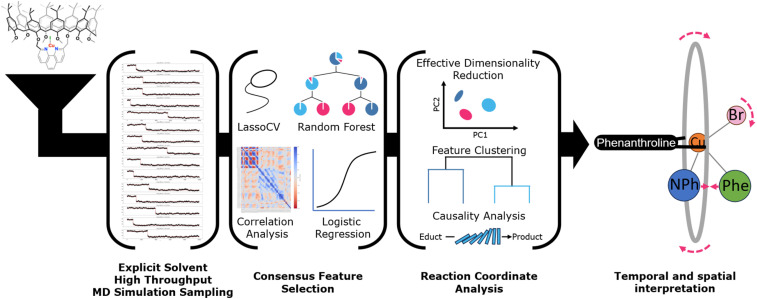
General workflow to identify and quantify the reaction coordinate by analyzing correlations and causality in an ensemble of reaction trajectories.

The multistep protocol developed to investigate the C–N coupling dynamics with the [Cu(C_8_PhenMe_6_)I] catalyst is highlighted in Fig. 10. It involves high throughput explicit solvent MD sampling of the reaction step, followed by machine learning analysis, where consensus features are extracted. These are utilized for qualitative and quantitative analysis of the reaction coordinate. As a last step, the time evolution of the system is considered by applying a causality model that allows to redefine the reaction coordinate as a sequence of individual movements of groups of atoms.

### Simulation protocol

For the QM/MM MD simulation, we chose the in-house developed *ab initio* quantum mechanical charge field (QMCF) molecular dynamics approach^80^ using the link-bond method to describe the bonds crossing between QM and MM.^81,82^ The simulation parameters were set up using the GAFF^83^ force-field,^84^ using the PyConSolv^19^ 1.0.0 tool, with default settings. For the geometry optimization, the PBE0 functional^85^ was used with the def2-SVP basis set^86^ and D3 dispersion corrections^87^ in implicit chloroform, using CPCM.^88^ The system was solvated in a cubic periodic box with 1708 chloroform molecules. For detailed information regarding the simulation parameters, see the SI. The 54 atoms at the centre of the calixarene cage were included in the QM zone (see SI Fig. S1). The quantum mechanical calculations were performed at two distinct levels. The semi-empirical method GFN2-xTB was utilized, providing a vast speed-up of the QM calculation. As semi-empirical methods require benchmarking,^89–91^ a full DFT reference was used, with PBE0/def2-SVP/D3, using Turbomole, showing very good agreement.^92^ A detailed comparison can be found in the SI (Section 4).

We used chloroform in our simulations instead of toluene, which was chosen experimentally. Chloroform and toluene have comparable permittivities (*ε*_r_ 4.8 and 2.4),^93^ both typical of apolar solvents. Thus, both solvents provide similar apolar environments, consistent with the observation that the catalyst is active in different media. Experimentally, the reaction also works in tetrachloroethane but toluene was selected as a more benign and environmentally safe option. As there is no evidence for strong solvent effects on reactivity, the choice of solvent is unlikely to significantly alter the system. In simulations, chloroform was preferred because its smaller size enables faster equilibration than toluene.

To generate an appropriate starting structure the system was equilibrated and a 100 ns of a MM/MD simulation was carried out, using the multistep protocol implemented in PyConSolv.^19^ We conducted 152 simulation runs using the QM/MM MD protocol of the reductive elimination step, employing GFN2-xTB as the QM method. Among these runs, 142 simulations captured the reductive elimination step and were utilized for evaluating the reaction barrier and structural analysis of the reaction. All 152 trajectories were used for labelling and evaluation of the reaction energy (see below).

### Ensemble labelling and reaction energy

From the MD simulation trajectories, cartesian coordinates and QM energies of the catalyst were extracted. The QM energy was used to label the individual frames as educt, transition state and product, while employing a filter function to minimize random fluctuations. The transition states were identified as the frames that define the last energy maximum before the large drop in energy associated with the formation of the product (see SI Section 2 for details), that is, all frames before the TS region were considered as educt, all frames following the TS region as product. To assess whether this is a viable labelling of the states, we analysed the distribution of internal coordinates in Fig. S5, where we can see a clear separation of educt, transition state, and product for example in the decisive C–N coordinate, but also in the Phe–N bond length distribution.

The reaction barrier was defined as the difference between the transition state structure energy and the maximum value of a sigmoid function fitted through the reaction profile (see Section 2 of the SI). As DFT sampling on a similar scale as GFN2-xTB was not achievable, the ensembles were too small to obtain the reaction energy by averaging over them. Thus, to evaluate the reaction energy from the DFT simulations, we fitted sigmoids between the educts and product. By subtracting the highest sigmoid value from the lowest we were able to calculate a reaction energy and compared it to that obtained from GFN2-xTB using the same approach. As further confirmation of the accuracy of the transition state labelling, we confirmed that the structures in the ensemble resemble that of a transition state optimized using eigenvector following in a static approach (see SI Section 4 and Fig. S3).

### Identification of the reaction coordinate

We resorted to different coordinate systems to describe the reaction coordinate. We generated a set of fully redundant internal coordinates for all trajectories, using the MDanalysis package.^94^ This has the advantage of removing the issue of noise due to alignment artefacts, yet introduces more correlational effects.^76^ We also generated a reduced set of internal coordinates, describing highly rigid chemical moieties by their centre of mass (see SI Section 5 for details).

We utilized Principal Component Analysis (PCA)^37^ and Time-lagged Independent Component Analysis (tICA)^38^ as dimensionality reduction techniques to identify coordinates that separate the states. The two unsupervised methods complement each other in regards to addressing the variances present in the dataset.^95,96^ For supervised dimensionality reduction, we opted for Linear Discriminant Analysis (LDA)^77^ due to its efficacy in separating distinct classes within a given dataset. To further enhance the separation capability of PCA and LDA, several methods of automated feature selection were chosen and implemented, namely Recursive Feature Elimination with Cross Validation^97^ (RFECV) using both Random Forest^98^ (RF) and Logistic Regression (LR)^99^ as classifier models, and Lasso^100^ with Cross Validation (LassoCV), all using fivefold stratified cross-validation.^101^ The RF and Logistic Regression classifiers were additionally evaluated by selecting only the top 10% of features. When subjecting the highlighted features to dimensionality reduction using PCA or LDA, we observed varying importance of the features to the principal components (SI, Tables S3 and S4). To alleviate this discrepancy between the results and increase separation performance, we defined a consensus model, which combines the results of all previously mentioned approaches and highlights features that are consistently found across all models. These features were identified by performing a PCA and LDA on each of the models and examining the loadings. Subsequently, we computed the average contributions to the first three principal components, as well as the two LDA components. The selected features needed to appear in at least 75% of the elimination models to be deemed significant. This process yielded a set of 49 internal coordinates for the PCA. These coordinates were hierarchically clustered to group together correlated movements^102^ and then subjected to causal inference analysis (see statistical model for causality inference).^102^

In tandem with automated feature reduction methodologies, we leveraged Decision Trees to quantify the most relevant features from our dataset. These Decision Trees underwent training with class balancing that is augmenting the ensembles *via* oversampling. Furthermore, we adopted a Decision Rule framework, employing the skoperules^103^ library. Within this framework, a Random Forest bagging classifier, consisting of 30 Decision Trees, was used to provide a semantically quantitative characterization of the three ensembles.

### Statistical model for causality inference

We aimed to infer the cause of the onset of the chemical reaction. We eliminated trajectories with few educt structures (less than 100), thus having a total of 139 reaction sampling events. To infer Granger Causality^74^ (GC) we followed the protocol outlined by Toda and Yamamoto.^75^ This involved performing the augmented Dickey–Fuller^104^ and Kwiatkowski–Phillips–Schmidt–Shin^105^ tests to ascertain the stationarity of the various time series. Most of the time series were deemed stationary, with only a couple of the features presenting non-stationarity, which were rendered stationary through differencing (see GitHub for the critical and test statistics for each test and trajectory).^106^ A multivariate vector autoregression model (VAR)^107,108^ was constructed and fitted with lag times varying from 0 to 50, for each time series. The appropriate lag time was chosen for each trajectory, based on the Akaike information criterion.^107,109^ The correlated time series were checked for cointegration using the Johansen test.^110^ Finally, we calculated a GC matrix for each feature, for every simulation, resulting in a total of 139 matrices, using VAR models trained at the appropriate lag time. To account for the occurrence of false positives with repeated sampling of the reaction, we applied the false discovery rate (FDR) correction proposed by Benjamini and Hochberg,^111^ with a threshold alpha of 0.1. The threshold for the GC test was set to *p* < 0.05. The full *p*-values for the causality matrices, non-FDR corrected, can be found on GitHub, along with the results of all statistical tests.

### Limitations of Granger causality statistical model

While the GC model has been shown to be a reliable approach for identifying causality, it suffers from some inherent limitations. The model requires the dataset to be presented in the form of time-series with constant variance and mean (stationary). To this end, the time-series stationarity must be verified and, depending on the results, rendered stationary through common statistical approaches such as differentiation. Another limitation in the nature of the GC analysis is the influence of hidden variables that may not have been taken into account in the initial dataset. This implies that the dataset must be carefully chosen, either manually curated or through automated means, such as feature selection. Moreover, the GC test also looks at first degree causality between the selected features, meaning that while we observe one variable influencing another, we cannot rule out that the influencing variable was not provoked by a 2nd variable beforehand. This can be overcome by performing a GC analysis on each possible pair of features and interpreting the results; however, this becomes difficult as the number of important features increases.

## Code availability

The python code used for analysis is made available on Github (https://github.com/PodewitzLab/MLReactCoord/releases/tag/v1.0.0) and the respective functions will be implemented in a future version of PyConSolv (https://github.com/PodewitzLab/PyConSolv) to facilitate a broad applicability.

## Author contributions

The project was conceived by R. A. T. and M. P., while I. C. provided chemical input for the system, required to devise the project. R. A. T. performed the QM/MM/MD calculations together with T. S. H., while J. G. implemented the rescaling barostat into the QMCF package, specifically for this project. R. A. T. devised the Jupyter Notebook to conduct the analyses. Analyses were edited by M. P. R. A. T wrote the original draft, M. P., T. S. H., and I. C. edited the draft. All authors agree with the final version of the draft. M. P. acquired the funding and supervised this project. The computational resources were provided by T. S. H and M. P.

## Conflicts of interest

There are no conflicts of interest to declare.

## Supplementary Material

DD-004-D5DD00216H-s001

## Data Availability

The *p*-values for the statistical analysis are provided on the supplies Github repository, alongside two example trajectories (https://github.com/PodewitzLab/MLReactCoord/releases/tag/v1.0.0). The full set of dry trajectory data used for analysis is provided on Zenodo under https://doi.org/10.5281/zenodo.14886709. Supplementary information: it contains the detailed simulation protocol, information on the labeling of data as well as statistics and performance of the methodology, robustness and performance of sampling, an overview over the distribution of all internal coordinates, details about the statistical analyses, detailed analysis of the product (distribution) and solvation effects. See DOI: https://doi.org/10.1039/d5dd00216h.
