# Supplementary material for: Coupling causality and interpretable machine learning to reveal the reaction coordinate of C–N coupling with a supramolecular Cu-calix[8]arene catalyst
Source: Digit Discov. 2025 Sep 2;4(10):2954–71. doi: 10.1039/d5dd00216h (PMC12421827; doi:10.1039/d5dd00216h)
Supplement: DD-004-D5DD00216H-s001 [file DD-004-D5DD00216H-s001.pdf]

Supplementary Information (SI) for Digital Discovery.
This journal is © The Royal Society of Chemistry 2025

Supporting Information for

Coupling Causality and Interpretable Machine Learning to Reveal the Reaction Coordinate of C-N Coupling with a Supramolecular Cu-Calix[8]arene Catalyst

R. A. Talmazan[1], J. Gamper[2], I. Castillo[3], T.S. Hofer*[2], M. Podewitz*[1]

[1] Institute of Materials Chemistry, TU Wien, Getreidemarkt 9, 1060 Wien (Austria), maren.podewitz@tuwien.ac.at

[2] Institute of General, Inorganic and Theoretical Chemistry, Leopold Franzens University of Innsbruck, Innrain 80/82, 6020, Innsbruck (Austria), t.hofer@uibk.ac.at

[3] Instituto de Química, Universidad Nacional Autónoma de México, Ciudad Universitaria, 04510, Ciudad de México (México)

Table of Contents

1. Simulation Protocol 3
2. Labelling of Data 4
3. Note on the Statistics of the Reaction Energies 5
4. Performance of GFN2-xTB vs. DFT 5
5. Coordinate Systems 6
6. Internal Coordinates Overview 7
7. Feature Elimination 15
8. Decision Trees 20
9. Product Analysis 21
10. Robustness and Convergence of Sampling 22
11. Solvation Effects 22
12. Product Interactions 23
13. Technical Note 26
References 27

1. Simulation Protocol

The simulation temperature was maintained using the Bussi-Donadio-Parrinello thermostat, set to keep the temperature at 298.15 K.[1] The velocity verlet[2]/RATTLE[3] algorithm was employed to integrate the equations of motion with a timestep of 2 fs, with all hydrogen atom bonds being constrained. The long-range electrostatic interactions were considered via the reaction field method[4] with a Coulombic cutoff of 12.5 Å.

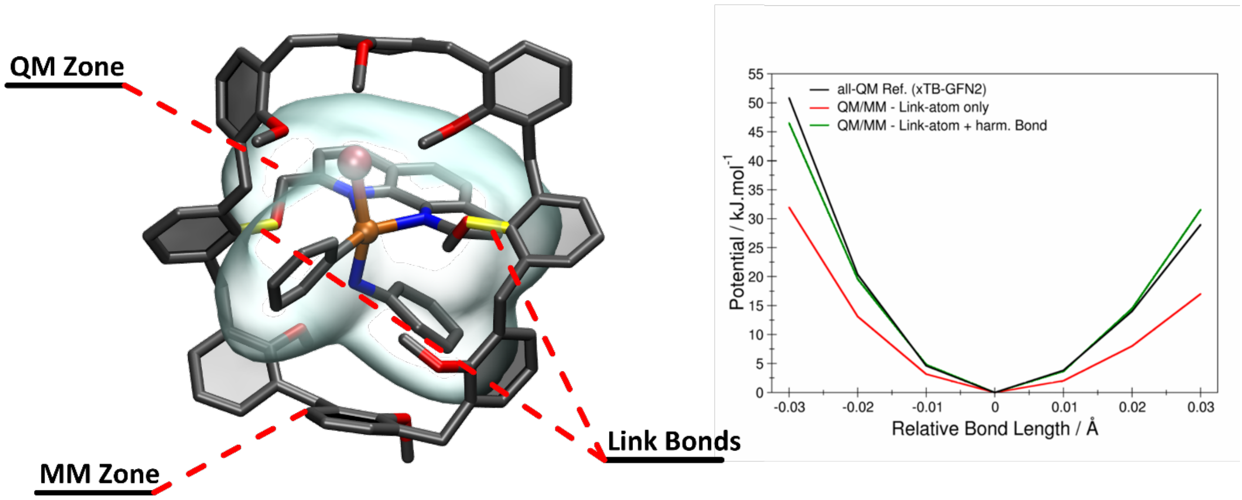

Figure S1. *The centre of the figure shows the QM and MM areas of the model system. The three potential curves belong to the bond stretching energy of the C-O link bond. The black curve represents the energy calculated at a full QM level using GFN2-xTB. The red curve represents the energy calculated for the QM/MM setup. The green curve is the result of applying a properly adjusted MM potential along the link-bond. It can be seen that the green and black curves are in very good agreement in the minimum region.*

A link bond approach was chosen to connect the quantum mechanical (QM) and the molecular mechanical (MM) region in the QM/MM calculation. The link bonds were defined between the oxygen atoms bonded to the phenanthroline bridge and the calixarene units 1 and 5 (see Figure S1). The parameters for the link bonds were calculated for both DFT and GFN2-xTB approaches (Table S1).

The QM/MM transition was facilitated by using the link-atom approach. The bond linking the oxygen atom of the phenanthroline bridge with the carbon of the calixarene unit was partitioned and the unsaturated valence was satisfied by placing a hydrogen atom at a position r_H on the vector defined by the frontier atoms of the QM and MM zones, determined by following formula:

$$r_H = \rho(r_C - r_O) + r_O \tag{1}$$

where ρ represents the ratio of distances for the bond length in the QM and MM representations, respectively. The position for the frontier QM oxygen atom is denoted as r_O, whereas the position of the frontier MM carbon atom is denoted as r_C.

To obtain the correct parameters for the link-bond, full QM and MM calculations of a model system, consisting of the QM zone (as seen in Figure S1) alongside calixarene units 2-6, were performed. In Figure S1, the energy of the bond stretching can be seen to be very similar for both the model system in the case of a full QM approach, as well as in the link-bond, when the appropriate harmonic potential is applied.

In Table S1 the resulting parameters of the link-bond (equilibrium bond distance r_{eq}, distance ratio ρ and the force constant K_{link}) can be seen for both methods.

Table S1. *Link bond parameters for the two methods. r_{eq} represents the equilibrium bond distance, ρ represents the ratio between QM and MM atom distances and K_{link} represents the force constant.*

**Method**	**r_{eq} (Å)**	**ρ_{link}**	**K_{link} (kcal/mol/Å^2)**
PBE0/def2-SVP/D3	1.3687	0.7124	335.786
GFN2-xTB	1.3866	0.7085	154.416

2. Labelling of Data

In order to label the structures, we extracted the QM energies from the reaction trajectories (Figure 2A). This energy comprised the potential energy of the QM zone, embedded in the electrostatic point charges of the solvent molecules. Due to simulation at finite temperature, random fluctuations, typically below 4 kJ/mol and categorised as noise, can be observed (Figure 2B). To enhance the clarity of reaction energy trends, we employed the Savitzky–Golay filter[5] with a bandwidth of 10 and a second-degree polynomial fit to smooth the reaction profile. Following this, data was then categorized into three distinct groups, educts (reactants), transition states, and products according to the following procedure: A sigmoid fit was performed through the energy trajectory as indicated in Figure 2C. After identifying the inflection point, an algorithm was used that goes backwards in the trajectory to identify the highest energy point and it stops once the energy drops again. The frame with the highest energy is identified as transition state. To account for structural noise, we also selected the frame immediately preceding and following the energy peak, resulting in a total of three structures per simulation run characterised as transition state region. All structures preceding the transition state are labelled educts, all states following the transition state are considered products (see Figure S2 A insert).

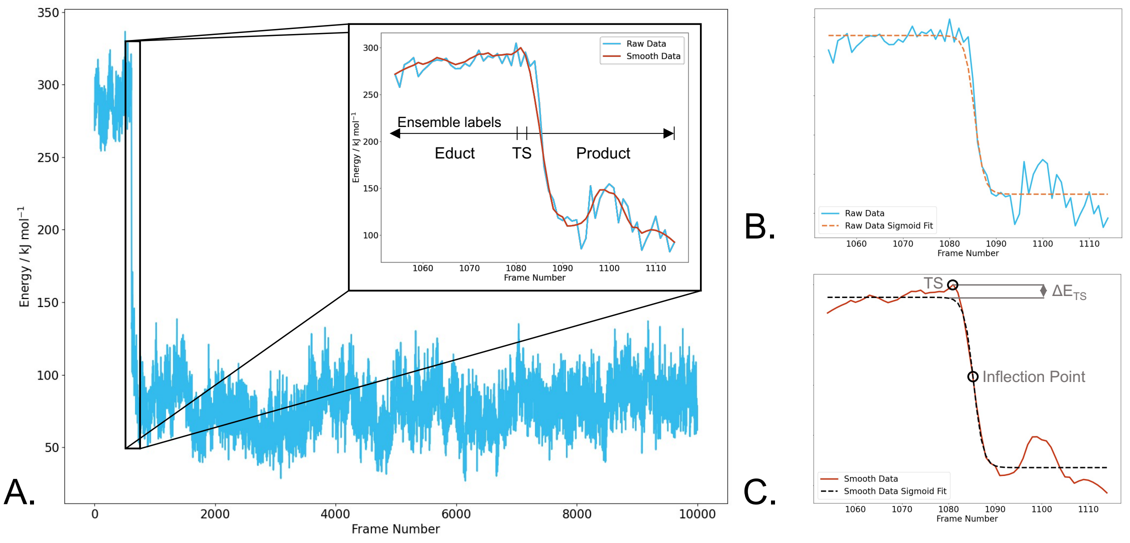

Figure S2. *Energy Diagram example of a QM/MM MD simulation of the C-N coupling step of phenylbromide and aniline using a Cu(I)-calix[8]arene catalyst. Depicted are energies of the QM region (plus MM point charges). A: Energy fluctuations for the whole duration of a simulation. Zoom focuses on the reductive elimination range in the simulation. The blue curve represents the raw energy data, the red curve represents the smoothed energy data. The black ranges in the pop-out rectangle indicate which labels have been assigned to the simulation frames; B: Energy curve and the sigmoid functions fitted to the raw reaction profile. Energies are for the QM zone, calculated with GFN2-xTB; C. Energy curve and the sigmoid function with inflection point and identified transition state region The TS barrier is identified as the difference between sigmoid fit and highest point, as indicated.*

These labels (Educt, TS and Product) were used for training the supervised machine learning models. To assess how reliable this procedure is the reader is referred to Section 6 of the SI.

3. Note on the Statistics of the Reaction Energies

The reaction energy reported in the main manuscript was calculated for the 152 trajectories by averaging the ensemble energy. The educt ensemble contained n = 412887 structures, the transition state ensemble contained n = 426 structures, while the product was constituted of n = 1106687 structures. The standard deviation was computed for each of the reaction states and propagated as the root of the sum of squared deviations of the states for the reaction energy calculation. For the transition state barrier, the same procedure was applied.

4. Performance of GFN2-xTB vs. DFT

We assessed the effect of smoothing and the performance of GFN2-xTB compared to DFT. As we do not have the same degree of sampling for DFT as for GFN2-xTB (8 vs 152 trajectories), we calculated the reaction energy by analysing 30 frames before and after the transition state and fitting a sigmoid function to the energy profile. However, as it is likely that the reaction energy is not fully re-distributed, we do not discuss the such calculated reaction energy in the manuscript, but only refer to the ensemble averaged energy. The reaction energy for this comparison is defined as the difference between the minimum and maximum values obtained from this sigmoid fit, while the reaction barrier corresponds to the difference between the transition state structure and the maximum value of the sigmoid function (See Figure S2). Both raw data and smoothed data are used to compute these energy values to estimate the effect of the smoothing function.

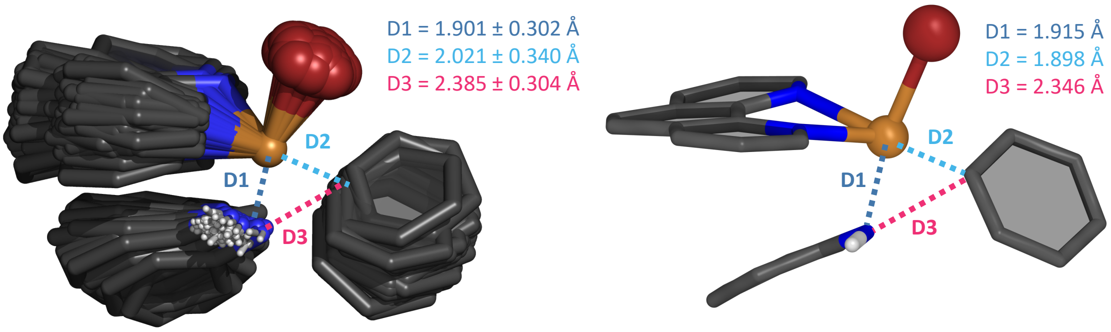

A. B.

Figure S3. *Comparison of transition state structures. A. structural ensemble extracted from GFN2-xTB simulations; B. structure from a static PBE0/def2-SVP/D3 study.*

We find that the GFN2-xTB reaction energies are in very good agreement with those computed with PBE0/def2-SVP/D3, as shown in Table S2, where the reaction energies differ within 4-6 kJ/mol and the barrier within 5-6 kJ/mol. Likewise, the structures obtained from GFN2-xTB are very comparable with those from DFT (Figure S3).

Table S2*. Reaction energies and barriers computed from the GFN2-xTB and DFT trajectories. All values are given in kJ mol^{-1}.*

E in kJ mol^{-1}		Mean	Median	Standard Deviation
Smooth GFN2-xTB	Reaction**	-165	-166	12
	Barrier	13	13	9
Raw GFN2-xTB	Reaction**	-163	-164	12
	Barrier	12	13	13
Raw DFT	Reaction**	-169		13
	Barrier	18		5

**The reaction energies reported here are obtained by sigmoid fit to compare GFN2-xTB and DFT results and cannot be compared to the numbers reported in the main manuscript that were obtained by ensemble averaging.

Based on this assessment, we believe that GFN2-xTB is accurate enough in this case, compared to PBE0/def2-SVP/D3. We believe this remarkable is due to the Cu(I) and Cu(III) nature of the investigated species. The validity of PBE0 was tested in previous studies, where it was found to be fully consistent with all experimental data.[6]

5. Coordinate Systems

We utilized different coordinate systems to assess the trajectories. By default, we used Cartesian coordinates, aligning all simulations on the rigid phenanthroline bridge to reduce rotational and translational noise.[7] We performed a PCA on the aligned Cartesian coordinates of the Cu(I)-calix[8]arene trajectories (Figure S4A), which allows separation of the data according to the largest variance and captures changes in system configuration.

As the calixarene system is highly flexible, there is considerable difficulty associated with performing a correct alignment of the simulation trajectories. This results in certain system-wide translational and rotational degrees of freedom remaining embedded in the cartesian coordinates. The PCA performance was lacklustre (Figure S4), where the 3 labelled classes cannot be properly separated. When investigating the Pearson correlation of the system coordinates (see Figure S4C), we observe signs of rotational motion, indicating excessive noise in the trajectory coordinates.

Another strongly correlated area in the matrix is represented by the reaction centre, which corresponds to the formation of the C-N bond and rearrangement of the atoms surrounding the copper.

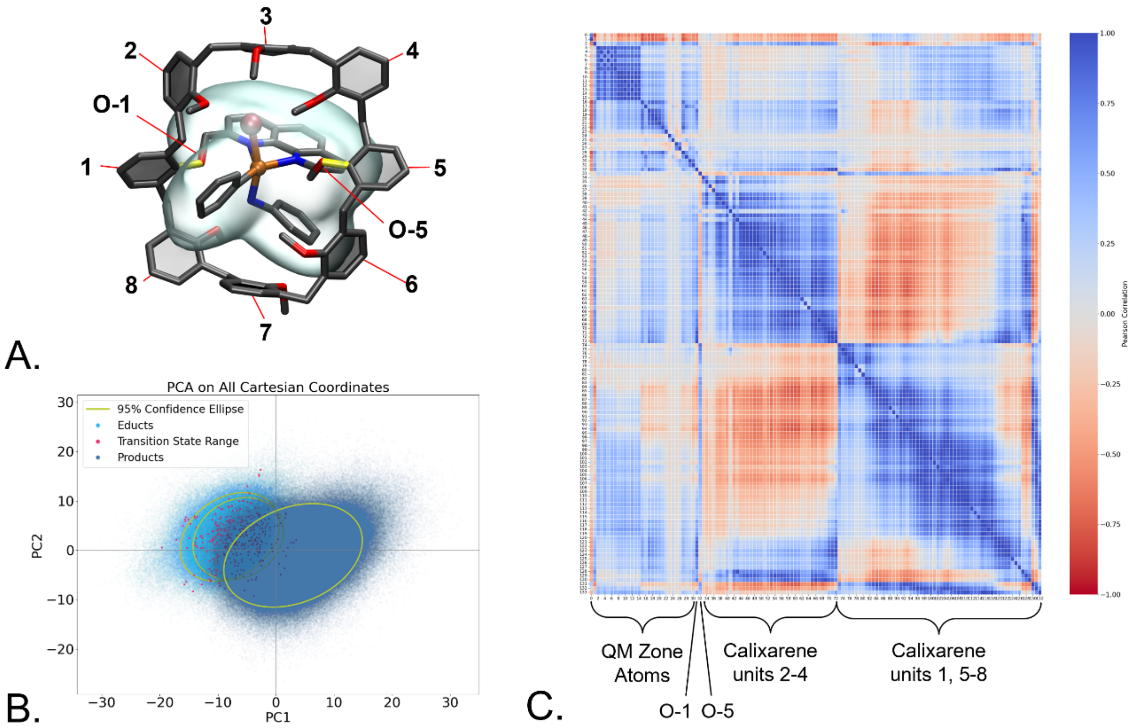

Figure S4. *A. Calixarene structure with the numbered calixarene units and oxygen atoms. The bubble surrounds the atoms in the QM zone of the QM/MM MD. B. PCA Analysis performed on the XYZ coordinates of all heavy atoms of the calixarene, on all data points across simulations. Colouring performed according to the ensembles: light blue: educt; magenta: transition state; dark blue: product. C. Pearson correlation matrix of the cartesian coordinates.*

To improve performance, we resorted to a highly reduced representation of the system. Specifically, we described each calixarene unit, the phenanthroline unit, as well as the phenyl and aniline moieties by their respective centres of mass only. Furthermore, we augmented the internal coordinates by incorporating critical individual atoms. This included oxygen atoms associated with the calixarene, as well as copper (Cu) and bromine (Br) atoms, along with carbon (C) and nitrogen (N) atoms participating in the coupling bond. The reduction in dimensionality comes with little loss of information, as the atoms in the highly rigid calixarene units always move together. All coordinate systems were normalised, using min-max normalization, to improve the performance of the classification models. No normalisation was used for the decision tree and decision rules approach, as we aimed to quantify the features which distinguish the three ensembles.

6. Internal Coordinates Overview

In the histogram plots below (Figure S5), the distribution of all reduced internal coordinates is shown, with calculated mean and standard deviation values for each ensemble.

By analysing the distribution of internal coordinates, we can identify three different categories: coordinates where all three ensembles overlap, for example, Phe-c5, Phe-c6, or Cu-Br, coordinates, where educt and transition state ensembles overlap completely and are separated from the products, for example, Cu-C, Cu-N, or Phena-Cu-Br, and coordinates where all three ensembles are completely or partially separated, for example, C-N and Phe-N (complete separation) or NPh-C (partial separation). In our view the overlap of educt and TS is indicative of an early transition state.

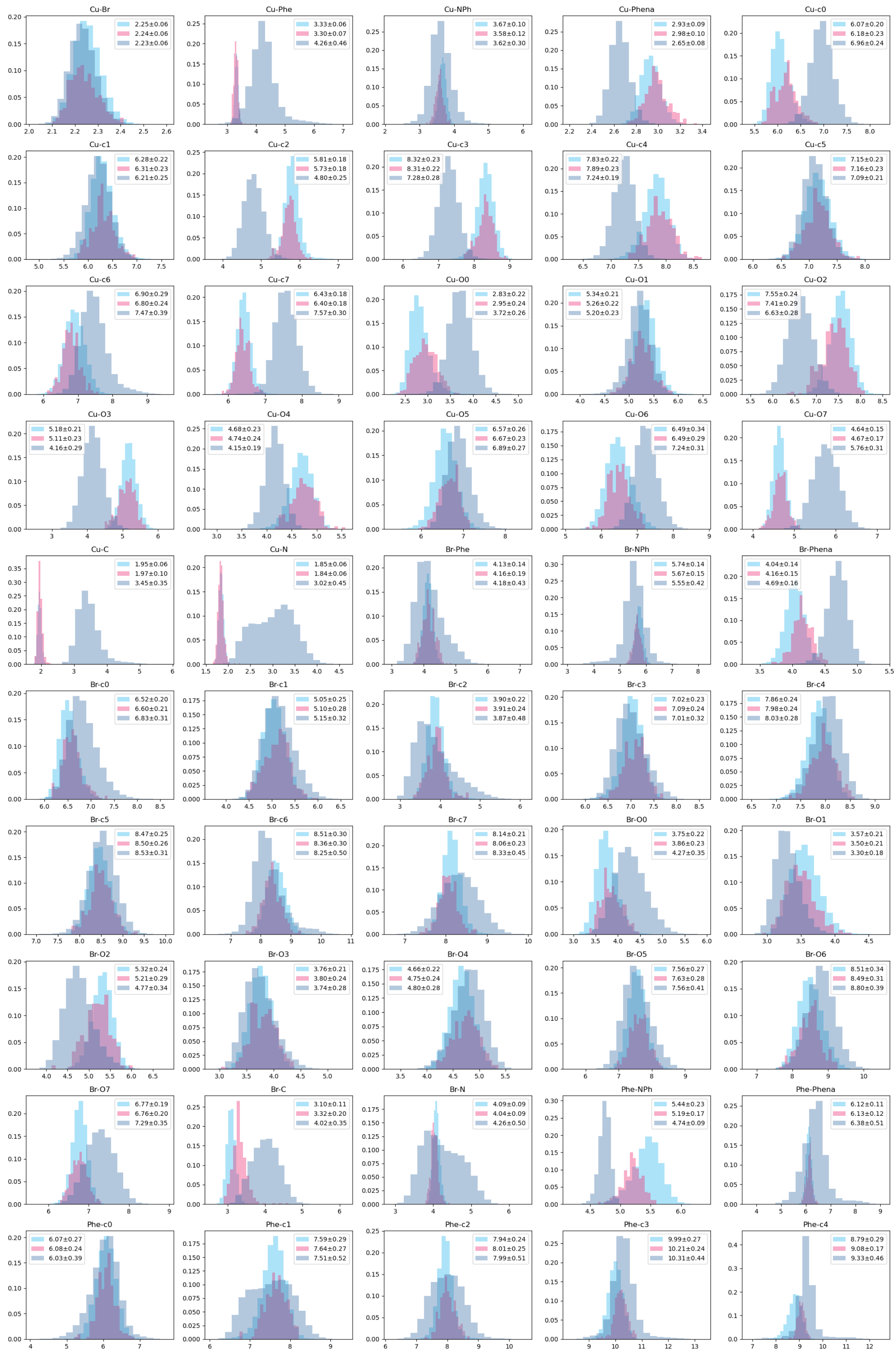

Figure S5. *Histogram plot of the distribution of internal coordinates for each ensemble with mean value and standard deviation. Light blue depicts the educt state, pink depicts the TS and dark blue depicts the product.*

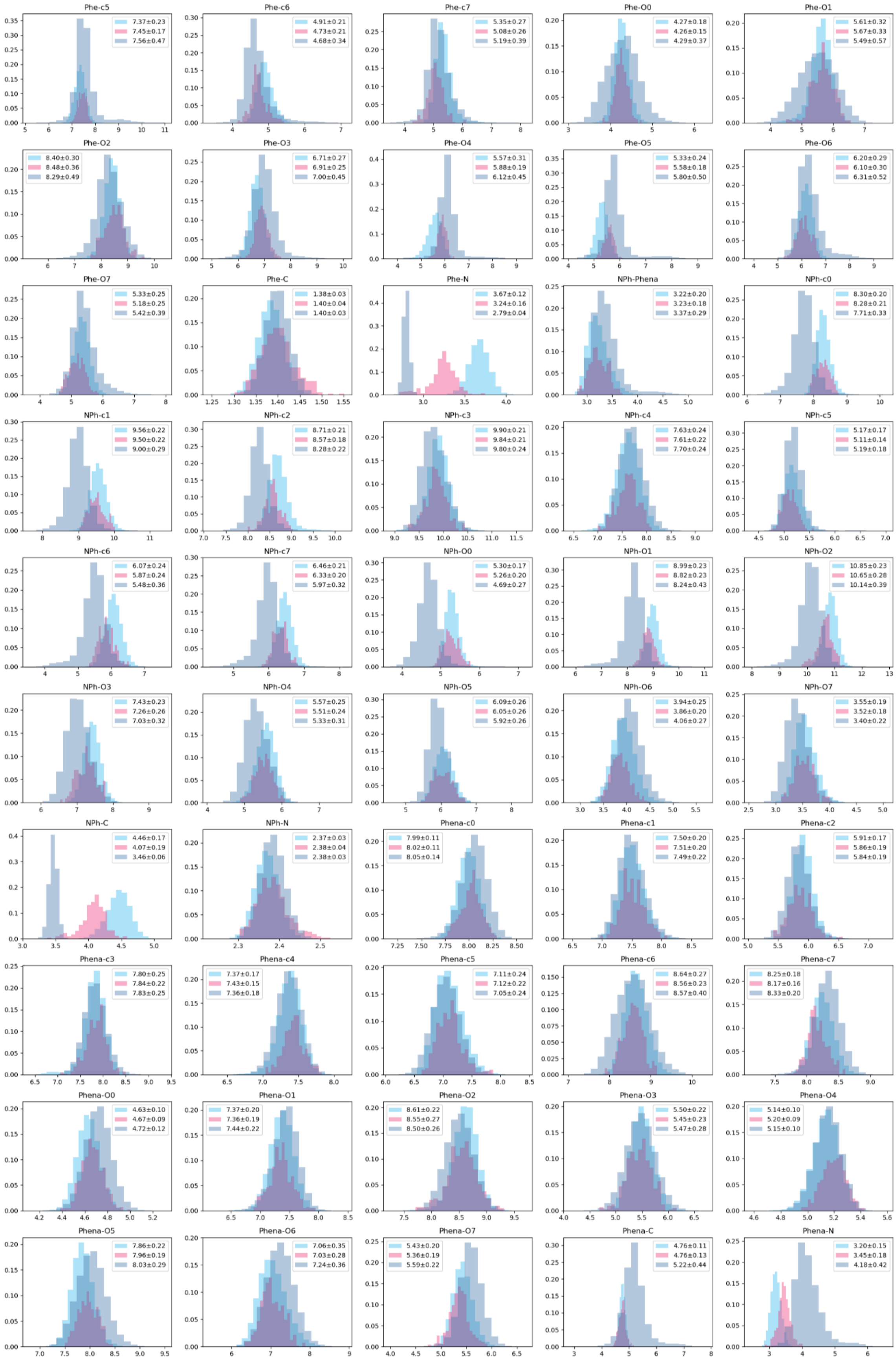

Figure S5 continued. *Histogram plot of the distribution of internal coordinates for each ensemble with mean value and standard deviation. Light blue depicts the educt state, pink depicts the TS and dark blue depicts the product.*

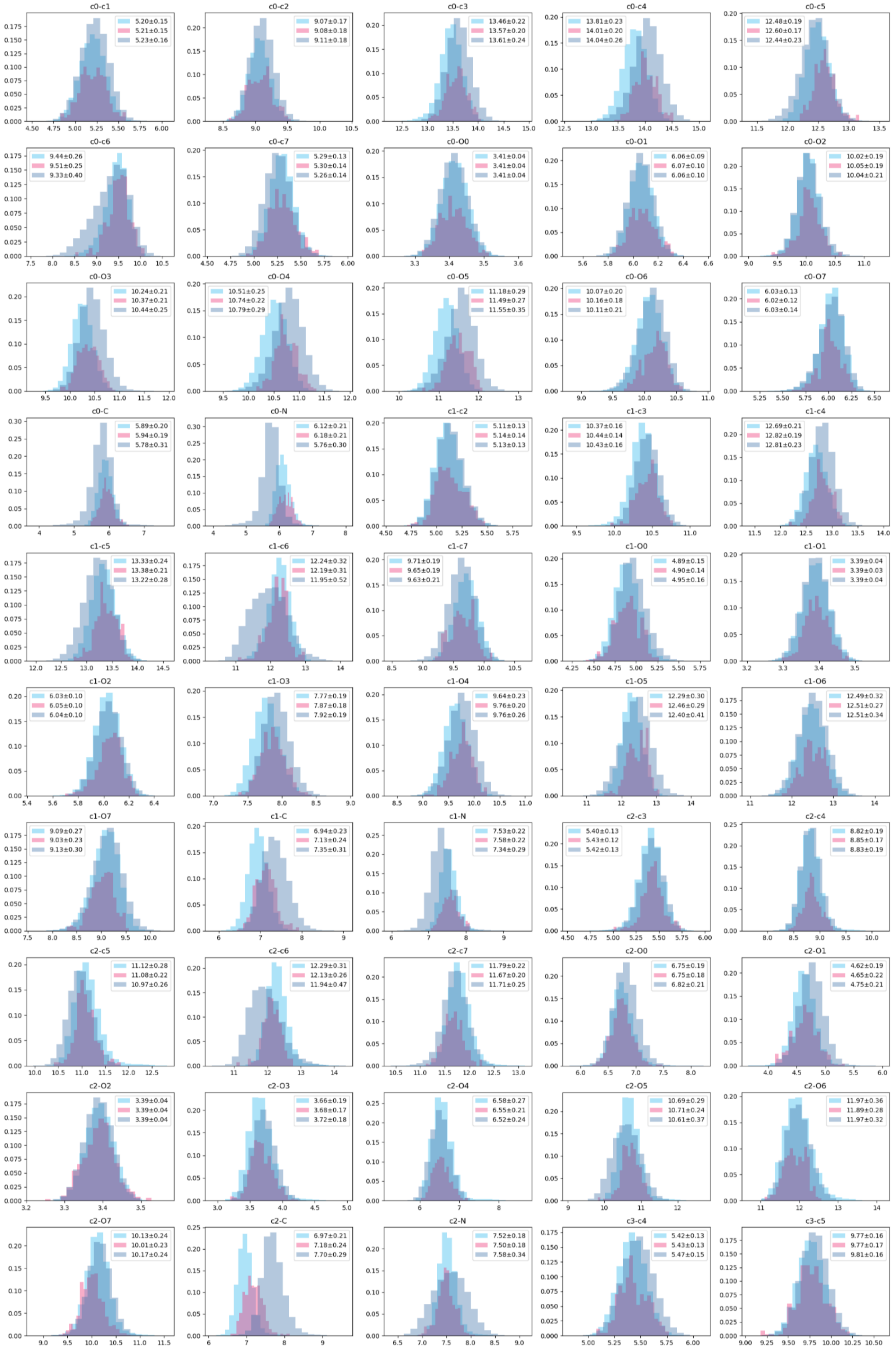

Figure S5 continued. *Histogram plot of the distribution of internal coordinates for each ensemble with mean value and standard deviation. Light blue depicts the educt state, pink depicts the TS and dark blue depicts the product.*

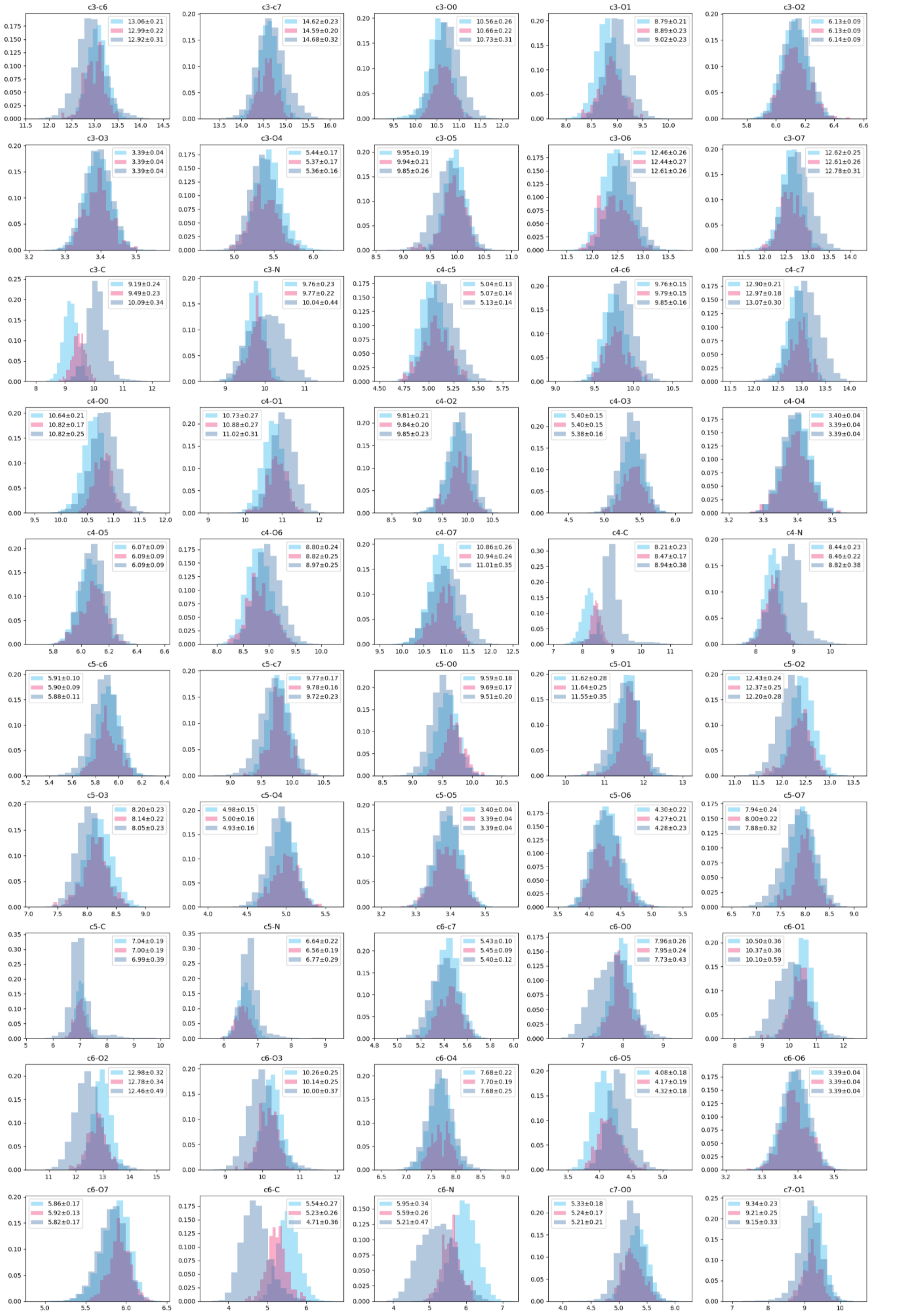

Figure S5 continued. *Histogram plot of the distribution of internal coordinates for each ensemble with mean value and standard deviation. Light blue depicts the educt state, pink depicts the TS and dark blue depicts the product.*

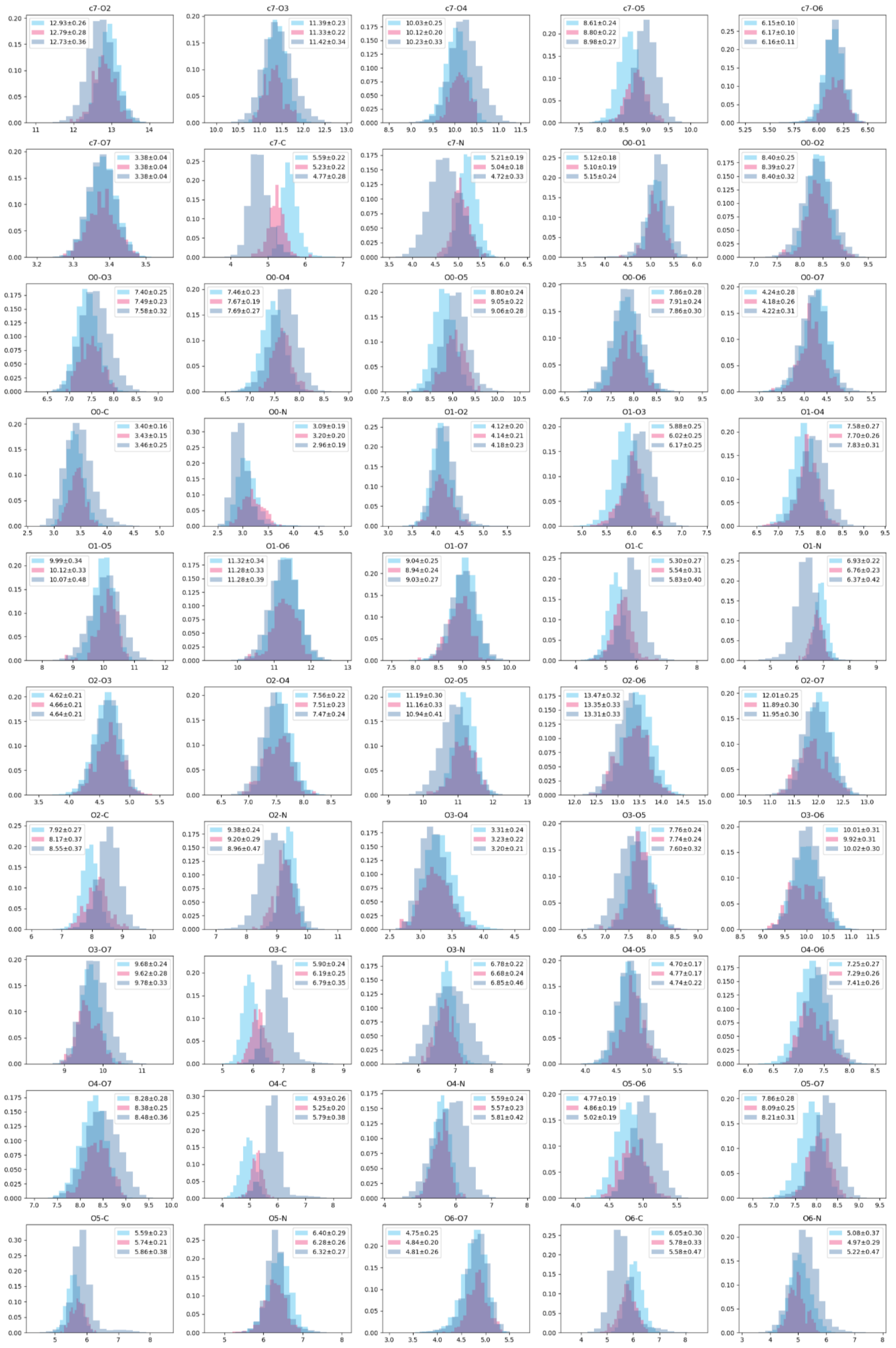

Figure S5 continued. *Histogram plot of the distribution of internal coordinates for each ensemble with mean value and standard deviation. Light blue depicts the educt state, pink depicts the TS and dark blue depicts the product.*

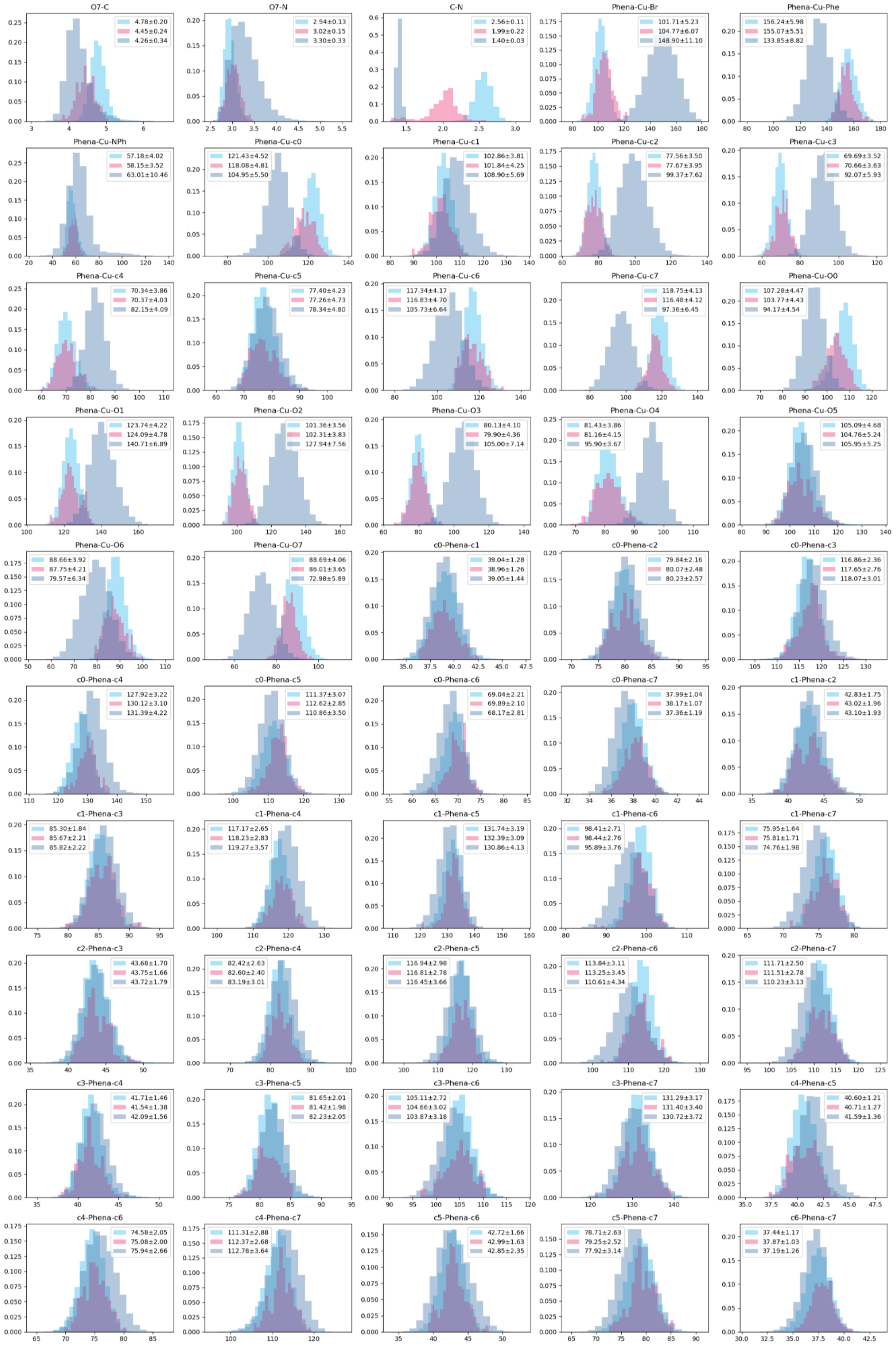

Figure S5 continued. *Histogram plot of the distribution of internal coordinates for each ensemble with mean value and standard deviation. Light blue depicts the educt state, pink depicts the TS and dark blue depicts the product.*

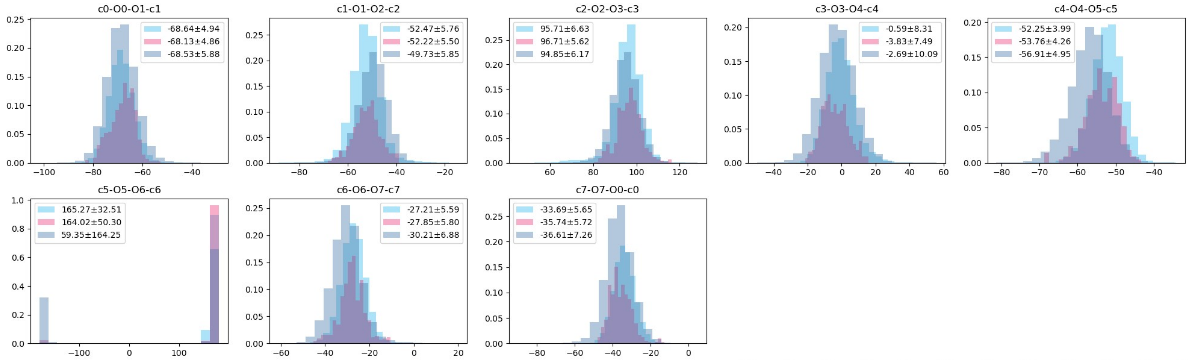

Figure S5 continued. *Histogram plot of the distribution of internal coordinates for each ensemble with mean value and standard deviation. Light blue depicts the educt state, pink depicts the TS and dark blue depicts the product.*

7. Feature Elimination

Using the ensemble labels derived from the reaction energetics, we were able to utilize supervised methods for feature reduction, in order to achieve a better separation of the reaction states. By feature reduction, we refer to a systematic elimination of internal coordinates, also known as features, which show no significant differences between the three ensembles. On the resulting reduced feature sets, we performed Principal Component Analysis (PCA, unsupervised) and, for sake of comparison, also Linear Discriminant Analysis (LDA, supervised) dimensionality reduction and evaluated the separation of the three ensembles, as well as the feature contributions to each principal component.

The feature elimination was performed using a fully manual approach, based on the correlation coefficient between the energy and features, a semi-automatic approach that involved training a classifier and using a cutoff for feature importance based on the mean of all feature importances, as well as a fully automated method. The latter involved automated recursive feature elimination with cross validation (RFECV) technique, with the random forest (RF) and logistic regression (LR) classifiers, and Lasso with cross validation (LassoCV). The feature importance for the models were extracted from the weights (Logistic regression) or mean impurity decrease indices (random forest).

While the manuscript only showed selected PCA dimensionality reduction results, the remaining results are shown in Figure S6 A, while the loadings are displayed in Table S3.

In addition to PCA dimensionality reduction, we also used feature selection for LDA. In LDA the feature selection technique had little to no effect on the ensemble separation (Figure S6 B).

When analysing the LDA loadings, the first principal component in LDA accounts for 99.9% of the dataset's variance (Table S4). Main contributing features are the changes in the distances around the catalytic centre and cage participants are dominating, with Cu-C, Cu-Phe and Phe-Phena.

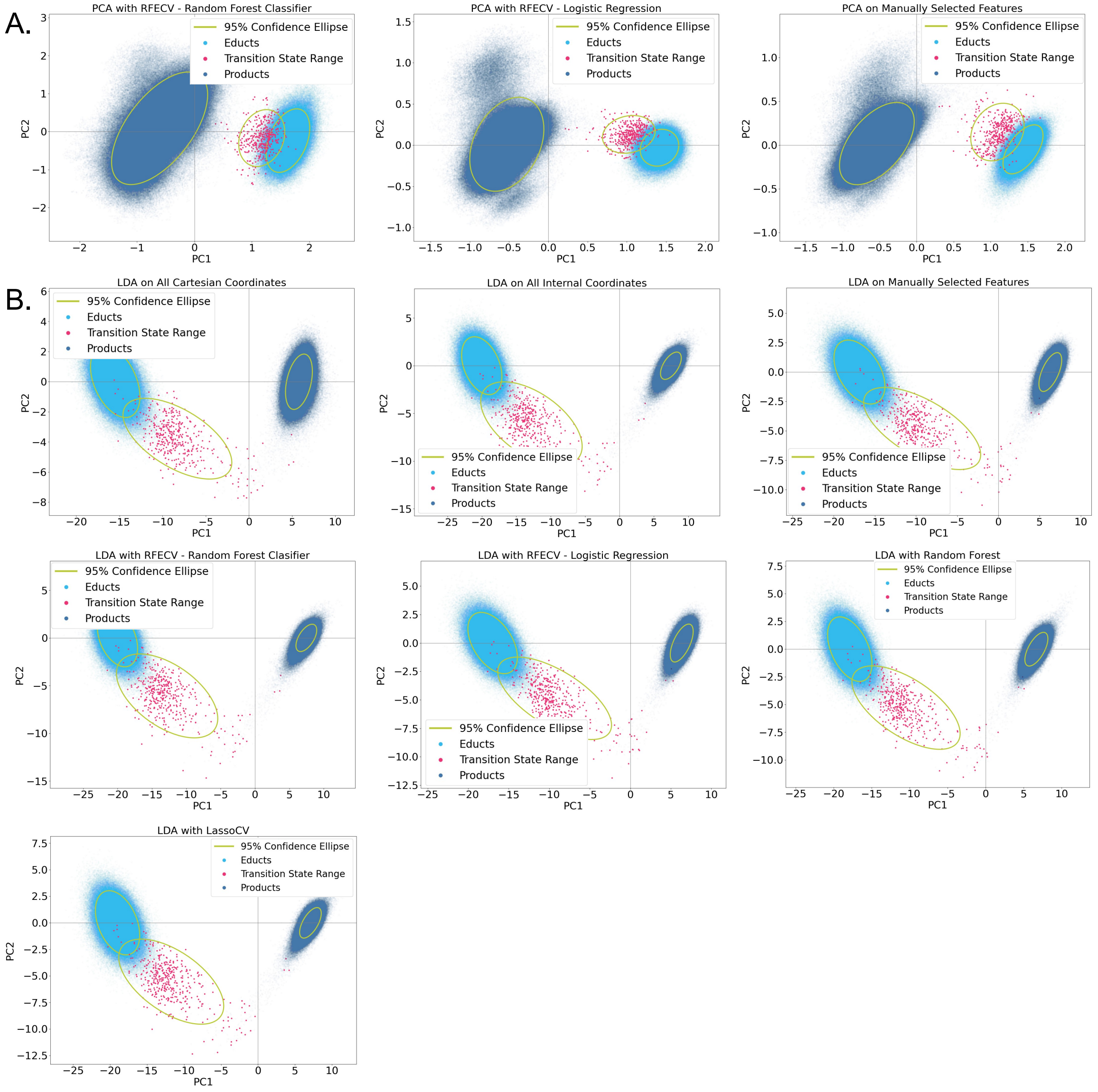

Figure S6. *A.PCA analysis of features selected by dimensionality reduction techniques as indicated in label. B. LDA analyses on the features selected by dimensionality methods as indicated in label.*

Table S3. Top 4 loadings of the PCAs with their normalized contributions, values in the brackets, to the respective principal component for each method.

Selection method	Principal Component	Coordinate (Normalized Contribution)				Variance
		1	2	3	4	
All internal Coordinates	PC1	C-N(1.00)	Phe-N(0.85)	Phena-Cu-Br(0.76)	NPh-C(0.76)	0.29
	PC2	c6-O0(1.00)	c1-c6(0.99)	Phe-c1(0.97)	c5-Phena-c6(0.88)	0.13
RFECV with Logistic Regression	PC1	c1-O1-O2-c2(1.00)	NPh-c3(0.85)	c4-O4-O5-c5(0.76)	Phena-c2(0.76)	0.42
	PC2	c3-O3(1.00)	Phe-c4(0.98)	c1-O2(0.87)	Phena-O6(0.86)	0.09
Random Forest	PC1	C-N(1)	Phe-N(0.85)	NPh-C(0.76)	Phena-Cu-Br(0.76)	0.68
	PC2	Phena-Cu-NPh(1.00)	Phena-C(0.98)	Cu-Phe(0.93)	Br-NPh(0.89)	0.08
Logistic Regression	PC1	C-N(1.00)	Phena-Cu-Br(0.77)	Phena-Cu-O2(0.62)	Cu-O7(0.62)	0.47
	PC2	Br-N(1.00)	O4-O7(0.98)	c4-O7(0.95)	O6-N(0.86)	0.10
RFECV with Random Forest	PC1	C-N(1.00)	Phe-N(0.85)	Phena-Cu-Br(0.76)	NPh-C(0.76)	0.32
	PC2	c6-O0(1.00)	c1-c6(0.98)	Phe-c1(0.96)	Phena-c6(0.89)	0.12
LassoCV	PC1	C-N(1.00)	Phe-N(0.85)	Phena-Cu-Br(0.76)	NPh-C(0.76)	0.35
	PC2	c1-c6(1.00)	Phe-c1(0.98)	c0-c6(0.87)	Phena-c6(0.86)	0.13
Consensus**	PC1	C-N(1.00)	Phe-N(0.85)	Phena-Cu-Br(0.76)	NPh-C(0.76)	0.76
	PC2	Phena-Cu-NPh(1.00)	Phena-N(1.00)	Cu-Phe(0.93)	Br-NPh(0.88)	0.06

**The values were obtained from the consensus model of both PCA and LDA.

Table S4. Top 4 loadings of the LDAs with their normalized contributions, values in the brackets, to the respective principal component for each method.

Selection method	Principal Component	Coordinate (Normalized Contribution)				Variance
		1	2	3	4	
All internal Coordinates	PC1	Cu-Phe(1.00)	Cu-C(0.93)	Phe-Phena(0.89)	O2-C(0.86)	0.9999
	PC2	Cu-O6(1.00)	Phena-O6(0.91)	Phena-Cu-c5(0.87)	Phena-Cu-O6(0.80)	0.0001
RFECV with Logistic Regression	PC1	Cu-Phe(1.00)	Cu-C(0.86)	Phe-Phena(0.85)	Phena-C(0.55)	0.9999
	PC2	Cu-C(1.00)	c1-C(0.89)	Cu-O7(0.83)	Phena-O7(0.83)	0.0001
Random Forest	PC1	Cu-Phe(1.00)	Cu-C(0.97)	Phe-c4(0.64)	Phe-NPh(0.64)	0.9999
	PC2	Cu-C(1.00)	Cu-Phe(0.91)	Phe-c4(0.58)	Phe-NPh(0.51)	0.0001
Logistic Regression	PC1	Phe-O6(1.00)	Cu-C(0.98)	Br-NPh(0.86)	O7-C(0.80)	0.9999
	PC2	Cu-C(1.00)	Cu-c6(0.76)	Br-NPh(0.66)	Cu-NPh(0.55)	0.0001
RFECV with Random Forest	PC1	Cu-Phe(1.00)	Cu-C(0.93)	Phe-Phena(0.88)	O2-C(0.86)	0.9999
	PC2	Cu-O6(1.00)	Phena-O6(0.92)	Phena-Cu-c5(0.87)	Phena-Cu-O6(0.80)	0.0001
LassoCV	PC1	Phe-Phena(1.00)	Cu-Phe(0.98)	Cu-C(0.90)	c5-C(0.89)	0.9999
	PC2	Phena-Cu-c5(1.00)	Cu-c5(0.90)	Cu-O6(0.86)	Phena-O6(0.80)	0.0001

When examining the feature contributions of both PCA and LDA principal components (Tables S3-S4, Figure S7), the results differ slightly, depending on the classifier which was chosen.

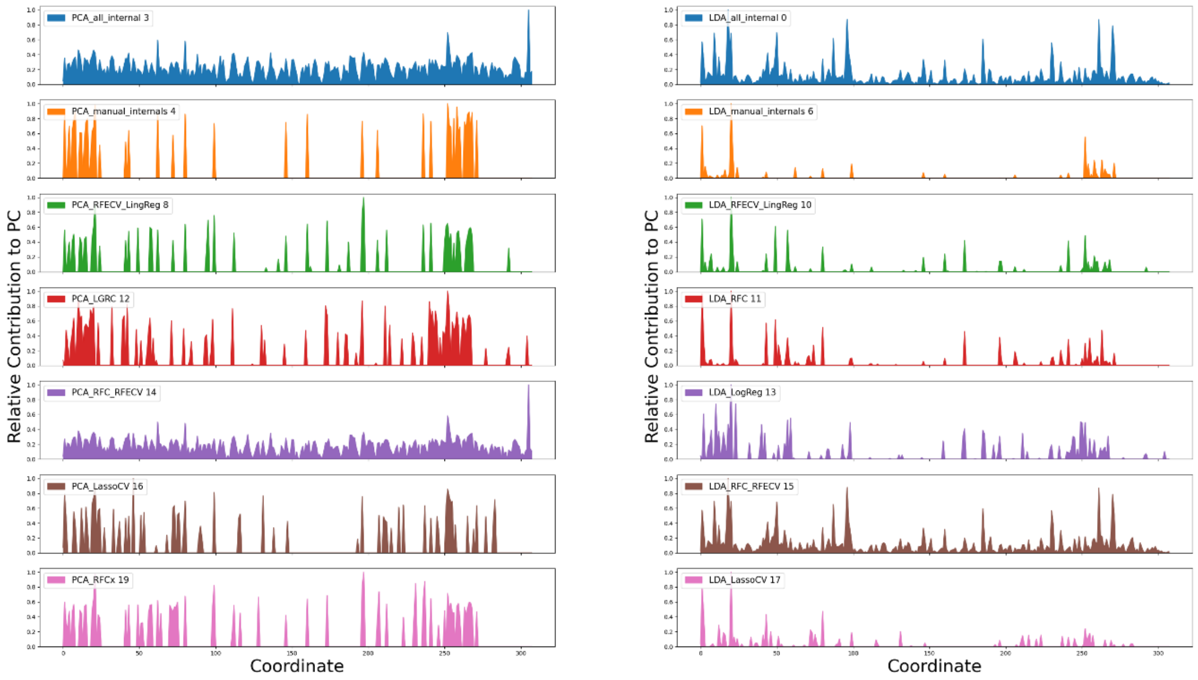

Figure S7. *Feature contributions from the various methods. The contributions are derived as the mean contribution to the principal components, PCA contributions are depicted in left graph, LDA contributions in right graph.*

In order to unite the individual features, a consensus model was developed that contained all features that were important in more than 75 % of all analyses (Figure S8). The consensus model evaluation of the internal coordinates revealed a large overlap between the PCA and LDA features. Hence, we decided to choose the final consensus model based on the feature contributions from both LDA and PCA, in order to maximise the performance in dimensionality reduction, using a best of both worlds type approach. The variance of this model is depicted in Table S3 and discussed in the main manuscript.

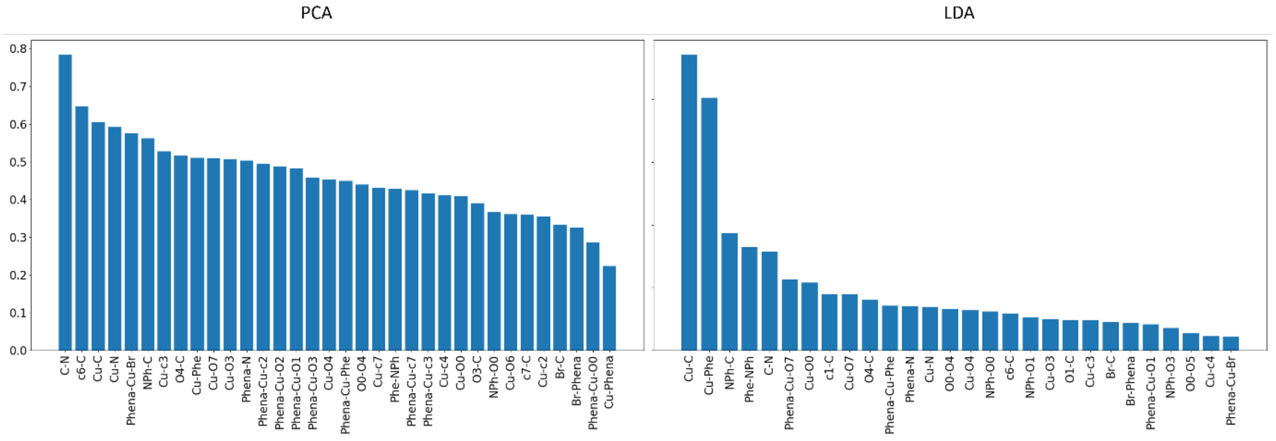

Figure S8. *High importance features from LDA and PCA consensus models.*

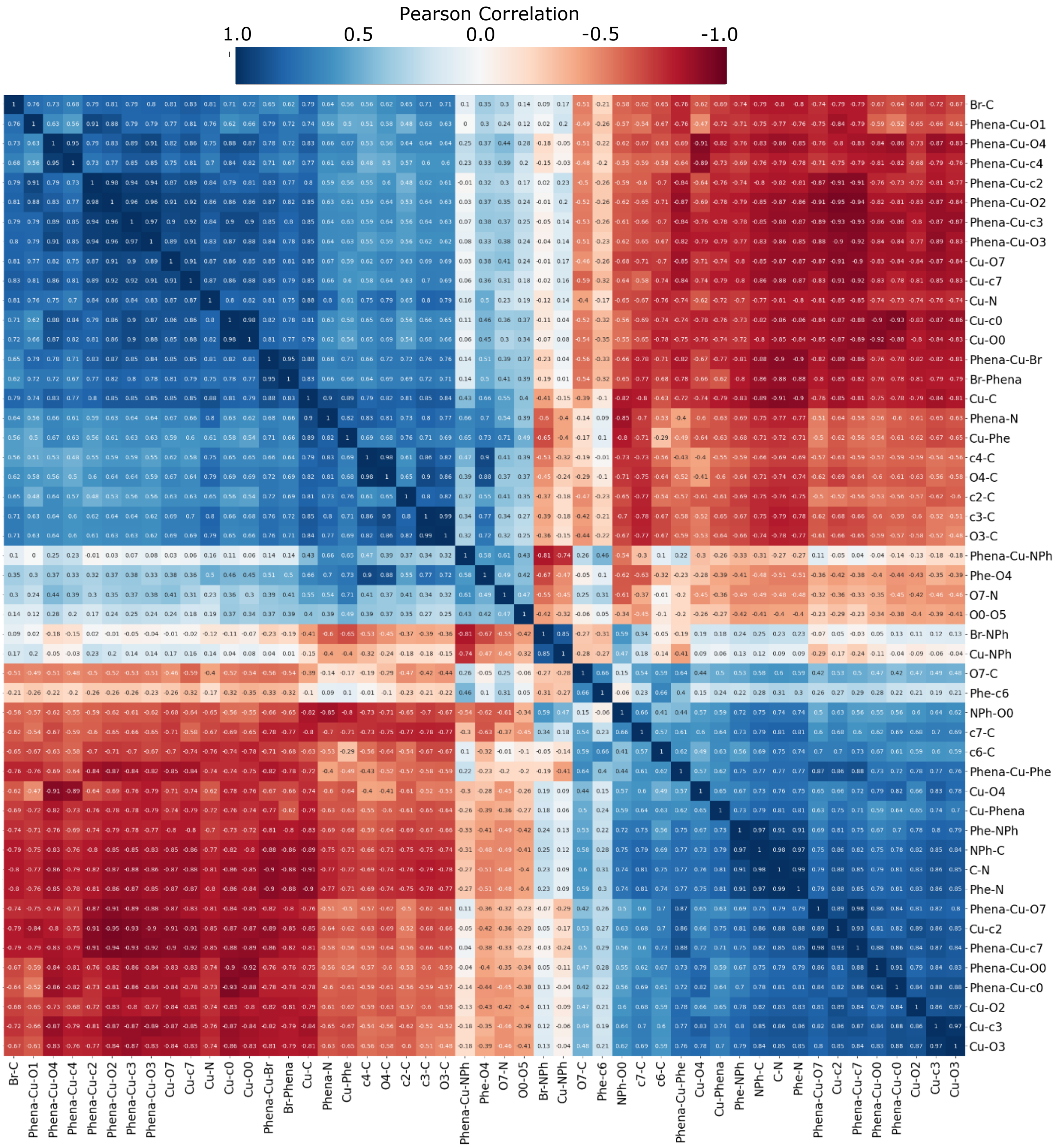

Figure S9. *Clustermap of the 49 features selected by the PCA consensus model.*

We obtained a clustermap (Figure S9) of the 49 important features by combining hierarchical clustering with the correlation matrix of the internal coordinates. We see two distinct regions of the complex with opposite correlation. Upon closer inspection of the individual features and their clustering, we can see the internal coordinates corresponding to the each calixarene unit group together. Coordinates that show positive correlation with the C-N bond (depicted in blue) decrease in value as the reaction proceeds, while those showing a negative correlation (depicted in red) increase during the reaction. Hence, the distances between Phe and NPh (comprising the product) and calixarene units 6 and 7 decrease (depicted in blue), indicating π-π stacking interactions, whereas C moves away from calixarene units 2-4 (coloured in red).

8. Decision Trees

This classifier separates the data based on precise cutoff values of the underlying features. The larger the tree structure, the more prone to overfitting it is. At the same time, the larger tree offers more detailed insight into what separates the structures. As we are not interested in performing predictions, an over-fitted tree is not of concern, but it's structure must be pruned, by limiting the size of the tree, in order to keep it interpretable.

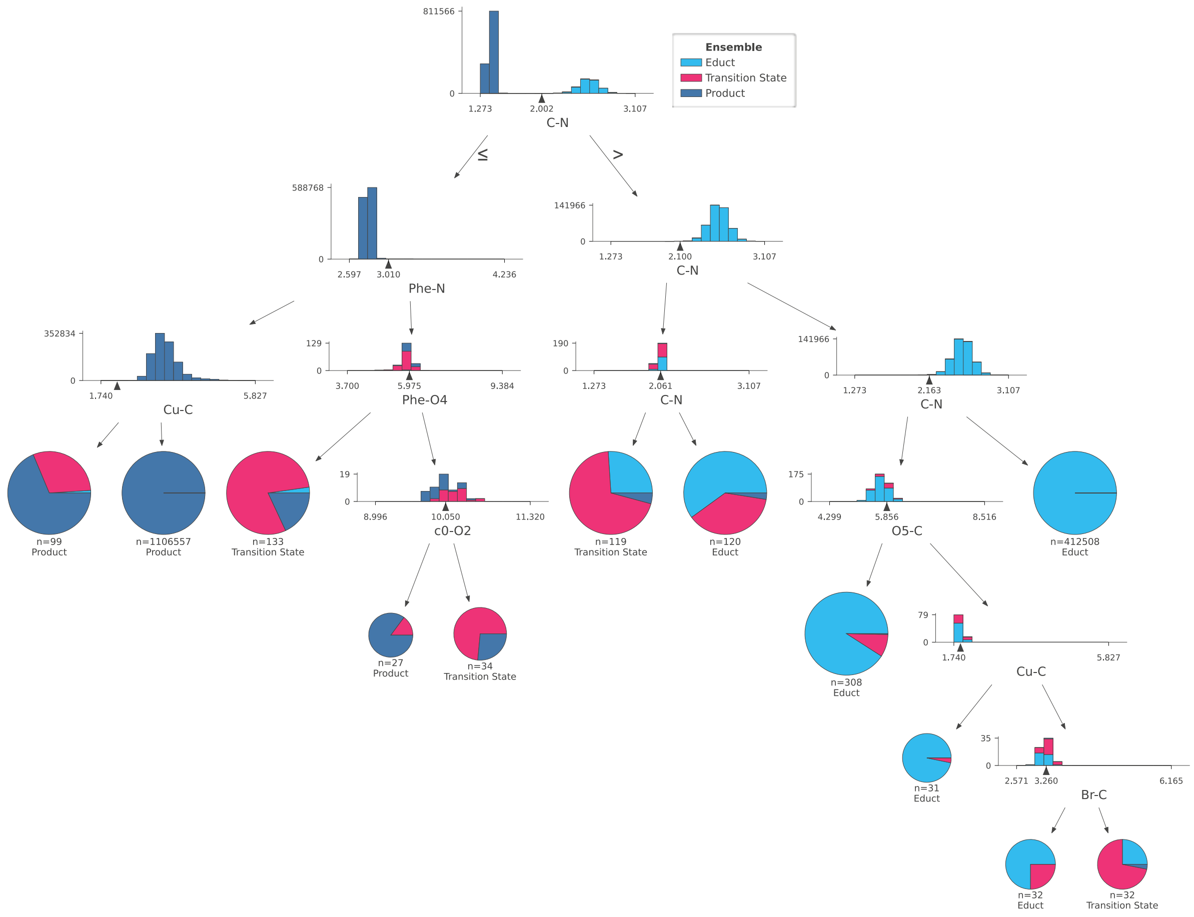

Figure S10. *Decision tree trained on the unbalanced dataset.*

When training the decision tree, it's important to ensure that the classes are balanced, as otherwise the class with lower representations might be disfavoured compared to the others. This was the case for our dataset, as the transition state ensemble contains only 426 structures, compared to the over 100000 for the other ensembles (Figure S10). To combat this, we used oversampling. By repeatedly drawing from each ensemble, we were able to equalize the number of data points between all ensembles. This way, each class used in the decision tree becomes equally important.

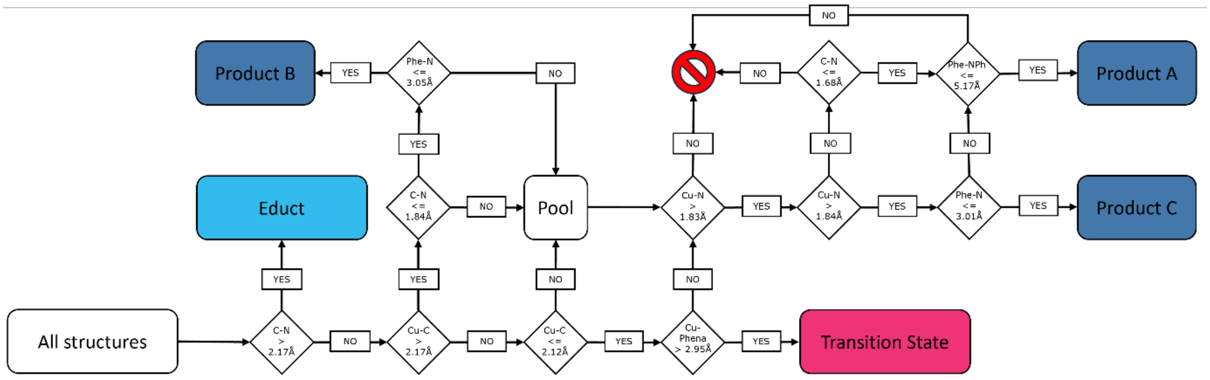

Figure S11. *Decision rules depicted in diagram form.*

The decision rule approach provides a semantic output of the decision tree, which can be transposed in a decision workflow as shown in Figure S11. The vast majority of structures can then be assigned to the appropriate ensemble, with only a few (less than 0.001%) not finding an assignment.

9. Product Analysis

To analyse the differences among the three product states defined by the decision rules and visible in the PCA with consensus features, the products were classified according to their respective decision rules. Color-coding these states in the PCA with consensus features demonstrated clear separation between them (Figure S12). This separation was further validated by k-means clustering, with the cluster centroids also shown in Figure S12. The distinction between the states is primarily attributed to PC2, as well as IC2 from the tICA analysis.

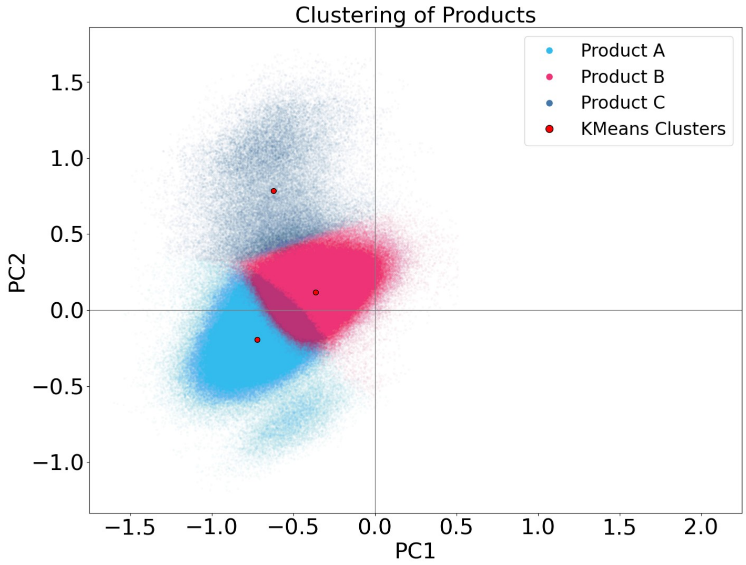

Figure S12. *Clustering of the product ensemble using K-means, projected in the consensus PCA space. Each cluster is coloured independently and the cluster centroids are displayed as red points with a black outline.*

10. Robustness and Convergence of Sampling

We evaluated the robustness of the sampling by performing dimensionality reduction on the consensus features using a reduced number of simulations and comparing it to the full dataset. We saw (Figure S13) that taking 10 random simulations from the dataset results in a PCA which resembles that of the full dataset, yet fails to distinguish between the product conformations.

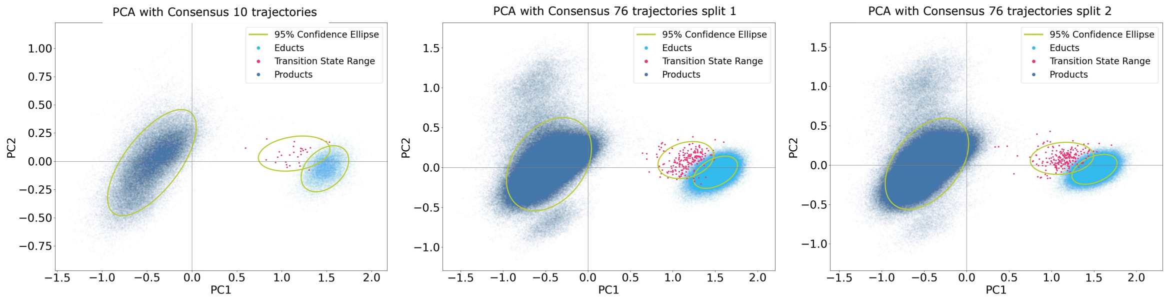

Figure S13. *PCA performed on the consensus features using 10 trajectories (left), 76 trajectories (middle) and the remaining 76 trajectories (right).*

When we randomly split the trajectories into two datasets with 76 runs each and perform the PCA, we see that they are very similar to each other in the separation of classes and product conformations. They strongly resemble the results of the PCA performed on the complete dataset. This indicates a convergence of the results and sufficient sampling of the reaction step.

11. Solvation Effects

The calix[8]arene system cavity has a tendency to collapse when calculations are performed with implicit solvation, as shown in Figure S14 (right). However, the cage has to stay intact to maintain catalytic activity. Consequently, explicit solvation is a requirement for a computational study in order to obtain accurate structures and energies of the system.

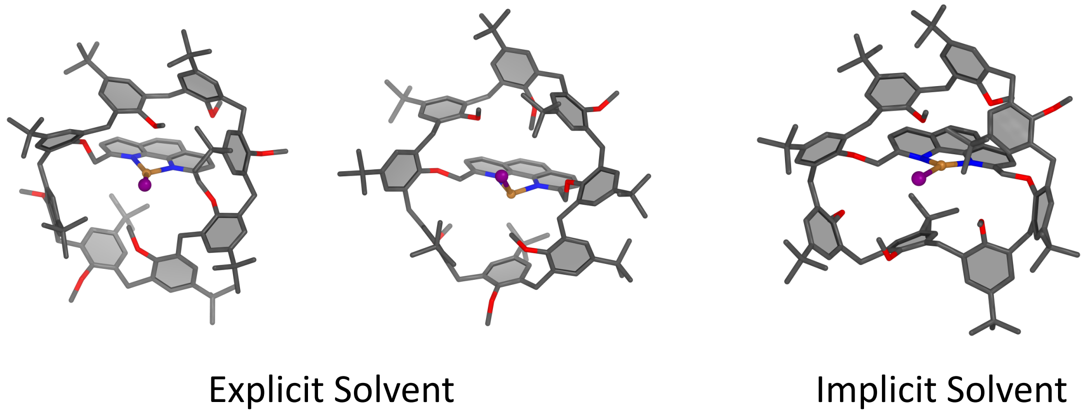

Figure S14*. Left: intact cavity when the system is solvated in explicit chloroform. Right: Collapsed cavity in the presence of implicit solvation.*

To validate the effects of the implicit solvent model on the calixarene cage, we performed two classical MD simulations (NVT ensemble, 300K, 1fs timestep with SHAKE enabled for hydrogen atoms), of the catalyst, in both implicit and explicit solvent. Notably, the cage for the implicit solvent system

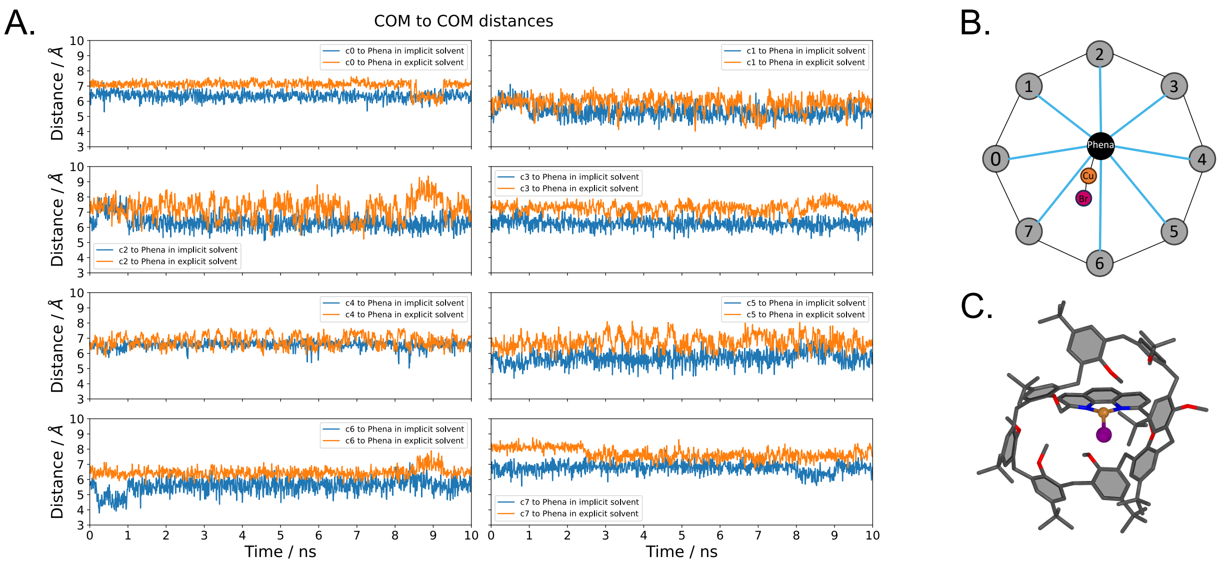

Figure S15*. A. COM to COM distances between the individual calixarene units and the Phenanthroline in both explicit and implicit solvent for a 10ns simulation. B. Schematic depiction of the COM to COM distances measured for the catalytic system. C. Simulated catalytic system.*

underwent a flip, exposing the catalytic centre, a phenomenon not observed in explicit solvent simulations. Such a behaviour would compromise catalytic activity. An implicit solvent model is therefore not appropriate to study this catalyst.

As a metric for defining the cage aperture, we chose the centre of mass of each calixarene moiety and measured the distance to the centre of mass (COM) of the phenanthroline. We only took into consideration the part of the simulation where the cage is still intact (10 ns). In the case of the implicit solvent simulation, the average distance was 6.10 ± 0.44 Å, while for the explicit solvent simulation, 6.91 ± 0.45 Å was measured. The implicit solvent simulation exhibits consistently lower values for the distances, as seen in Figure S15 and Table S5. This indicates that the cavity favours more compact conformations when simulated in implicit solvent, further highlighting the need for explicit solvation.

Table S5*. Individual calixarene to phenanthroline COM to COM distances in Å.*

	c1-Phena	c2-Phena	c3-Phena	c4-Phena	c5-Phena	c6-Phena	c7-Phena	c8-Phena
**Implicit solvent**	6.35 ± 0.26	5.33 ± 0.47	6.34 ± 0.48	6.25 ± 0.34	6.56 ± 0.25	5.70 ± 0.46	5.56 ± 0.50	6.76 ± 0.35
**Explicit solvent**	6.91 ± 0.54	5.94 ± 0.48	7.15 ± 0.56	7.36 ± 0.58	7.13 ± 0.30	6.71 ± 0.36	6.53 ± 0.44	7.57 ± 0.42
**Abs. Diff.**	0.56	0.61	0.81	1.11	0.57	1.01	0.97	0.81

12. Product Interactions

To further investigate and quantify the non-covalent interactions in the product, we clustered all product structures after alignment to the phenanthroline bridge with a Kmeans algorithm, based on the RMSD of the heavy atoms, obtaining five representative clusters. Please note that these clusters were not energy minimized but were taken as is.

<u>Methodology:</u> For each structure non-covalent interactions were calculated using a visualization of isosurfaces of the reduced density gradient as introduced by Johnson et al.[8] On these isosurface

regions the second eigenvalue of the Laplacian of the electron density is plotted. It allows distinction between strongly attractive forces (e.g., hydrogen bonds), weak attractions (e.g., van der Waals forces), and repulsive interactions (e.g., steric hindrance). The calculations were carried out with the NCIPlot tool,[9] which utilizes a wfn file as input. This file was produced via Turbomole,[10,11] employing the same density functional and basis set as those used in the ORCA calculations. Although different programs may yield slightly varying total energies even when applying identical methodologies, this does not pose a problem here. The focus is solely on the electron density, which is generally robust against such computational variations. The resulting noncovalent interaction maps were rendered using VMD.[12]

Results: The visualization of non-covalent interactions revealed weak dispersive interactions (indicated by the green coloring of the isosurface patches) between the phenanthroline bridge and the C-N coupling product as well as between the coupling product and the phenyl rings of the calixarene ligand. These are visible by the highlighted green patches in all five representative clusters (Figures S16-S19).

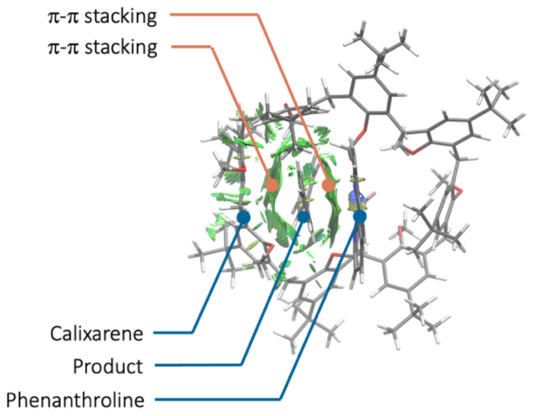

Figure S16*. Top view of the visualization of non-covalent interactions in* ***cluster representative 1*** *between the formed C-N coupling product and the phenanthroline bridge and the calixarene ligand, respectively. An isovalue of 0.05 a.u. was chosen for the isosurface of the reduced density gradient. Green values indicate weakly attractive interactions, such as van der Waals forces.*

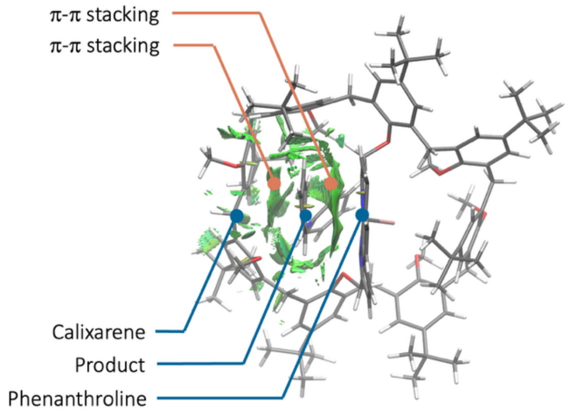

Figure S17. *Top view of the visualization of non-covalent interactions in* ***cluster representative 2*** *between the formed C-N coupling product and the phenanthroline bridge and the calixarene ligand, respectively. An isovalue of 0.05 a.u. was chosen for the isosurface of the reduced density gradient. Green values indicate weakly attractive interactions, such as van der Waals forces.*

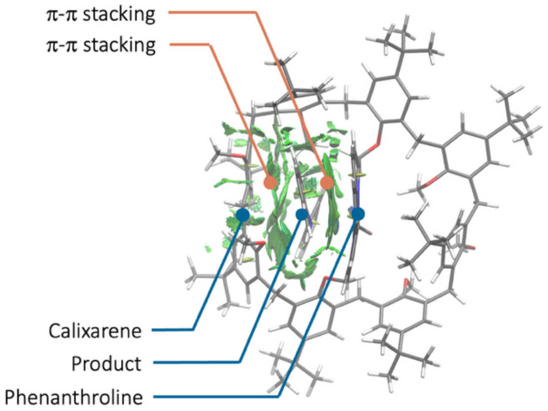

Figure S18. *Top view of the visualization of non-covalent interactions in* ***cluster representative 3*** *between the formed C-N coupling product and the phenanthroline bridge and the calixarene ligand, respectively. An isovalue of 0.05 a.u. was chosen for the isosurface of the reduced density gradient. Green values indicate weakly attractive interactions, such as van der Waals forces.*

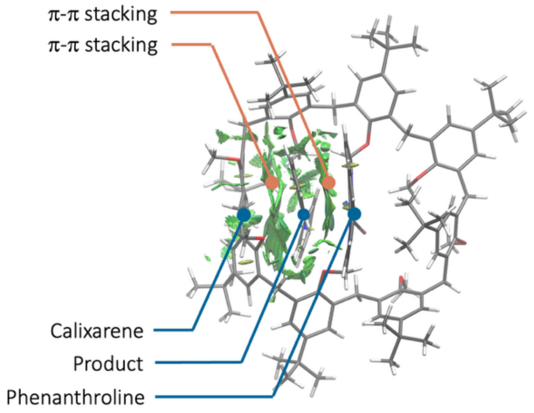

Figure S19. *Top view of the visualization of non-covalent interactions in* ***cluster representative 4*** *between the formed C-N coupling product and the phenanthroline bridge and the calixarene ligand, respectively. An isovalue of 0.05 a.u. was chosen for the isosurface of the reduced density gradient. Green values indicate weakly attractive interactions, such as van der Waals forces.*

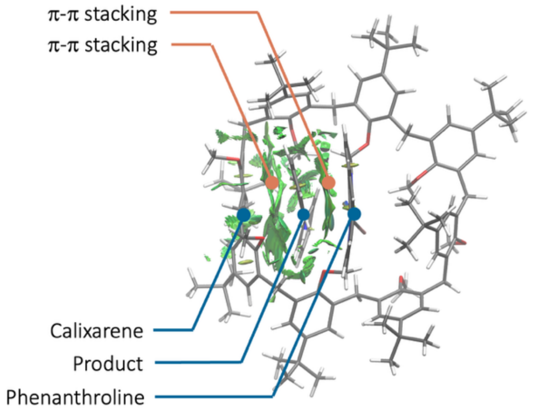

Figure S20. *Top view of the visualization of non-covalent interactions in* ***cluster representative 5*** *between the formed C-N coupling product and the phenanthroline bridge and the calixarene ligand, respectively. An isovalue of 0.05 a.u. was chosen for the isosurface of the reduced density gradient. Green values indicate weakly attractive interactions, such as van der Waals forces.*

13. Technical Note

Our implementation leveraged the following Python packages for data processing, statistical analysis and machine learning: pandas 2.2.1,[13] numpy 1.26.4,[14] scikit-learn 1.4.1.post1,[15] statsmodels 0.14.1,[16] scipy 1.12.0,[17] mdanalysis 2.7.0,[18,19] skoperules 1.0.1,[20] and pyemma 2.5.12.[21] For visualizations, we utilized dtreeviz 2.2.217,[22] seaborn 0.13.2,[23] and matplotlib 3.8.318.[24] The code used for analysis is made available as a jupyter notebook on Github (https://github.com/PodewitzLab/MLReactCoord) and will be integrated in the analysis suite of a future version of PyConSolv (https://github.com/PodewitzLab/PyConSolv).

References

(1) Bussi, G.; Donadio, D.; Parrinello, M. Canonical Sampling through Velocity Rescaling. *J. Chem. Phys.* **2007**, *126* (1), 014101. https://doi.org/10.1063/1.2408420.

(2) Swope, W. C.; Andersen, H. C.; Berens, P. H.; Wilson, K. R. A Computer Simulation Method for the Calculation of Equilibrium Constants for the Formation of Physical Clusters of Molecules: Application to Small Water Clusters. *J.Chem. Phys.* **1982**, *76* (1), 637–649. https://doi.org/10.1063/1.442716.

(3) Andersen, H. C. Rattle: A "Velocity" Version of the Shake Algorithm for Molecular Dynamics Calculations. *J. Comput. Phys.* **1983**, *52* (1), 24–34. https://doi.org/10.1016/0021-9991(83)90014-1.

(4) Barker, J. A.; Watts, R. O. Monte Carlo Studies of the Dielectric Properties of Water-like Models. *Mol. Phys.* **1973**, *26* (3), 789–792. https://doi.org/10.1080/00268977300102101.

(5) Savitzky, Abraham.; Golay, M. J. E. Smoothing and Differentiation of Data by Simplified Least Squares Procedures. *Anal. Chem.* **1964**, *36* (8), 1627–1639. https://doi.org/10.1021/ac60214a047.

(6) Talmazan, R. A.; Refugio Monroy, J.; del Río-Portilla, F.; Castillo, I.; Podewitz, M. Encapsulation Enhances the Catalytic Activity of C-N Coupling: Reaction Mechanism of a Cu(I)/Calix[8]Arene Supramolecular Catalyst. *ChemCatChem* **2022**, *14* (20), e202200662. https://doi.org/10.1002/cctc.202200662.

(7) Sittel, F.; Jain, A.; Stock, G. Principal Component Analysis of Molecular Dynamics: On the Use of Cartesian vs. Internal Coordinates. *J. Chem. Phys.* **2014**, *141* (1), 014111. https://doi.org/10.1063/1.4885338.

(8) Johnson, E. R.; Keinan, S.; Mori-Sanchez, P.; Contreras-Garcia, J.; Cohen, A. J.; Yang, W. T. Revealing Noncovalent Interactions. *J. Am. Chem. Soc.* **2010**, *132* (18), 6498–6506. https://doi.org/10.1021/ja100936w.

(9) Contreras-Garcia, J.; Johnson, E. R.; Keinan, S.; Chaudret, R.; Piquemal, J. P.; Beratan, D. N.; Yang, W. T. NCIPLOT: A Program for Plotting Noncovalent Interaction Regions. *J. Chem. Theory Comput.* **2011**, *7* (3), 625–632. https://doi.org/10.1021/ct100641a.

(10) Balasubramani, S. G.; Chen, G. P.; Coriani, S.; Diedenhofen, M.; Frank, M. S.; Franzke, Y. J.; Furche, F.; Grotjahn, R.; Harding, M. E.; Hättig, C.; Hellweg, A.; Helmich-Paris, B.; Holzer, C.; Huniar, U.; Kaupp, M.; Marefat Khah, A.; Karbalaei Khani, S.; Müller, T.; Mack, F.; Nguyen, B. D.; Parker, S. M.; Perlt, E.; Rappoport, D.; Reiter, K.; Roy, S.; Rückert, M.; Schmitz, G.; Sierka, M.; Tapavicza, E.; Tew, D. P.; van Wüllen, C.; Voora, V. K.; Weigend, F.; Wodyński, A.; Yu, J. M. TURBOMOLE: Modular Program Suite for Ab Initio Quantum-Chemical and Condensed-Matter Simulations. *J. Chem. Phys.* **2020**, *152* (18), 184107. https://doi.org/10.1063/5.0004635.

(11) *TURBOMOLE V7.5 2020, a development of University of Karlsruhe and Forschungszentrum Karlsruhe GmbH, 1989-2007, TURBOMOLE GmbH, since 2007; available from http://www.turbomole.com.*

(12) Humphrey, W.; Dalke, A.; Schulten, K. VMD: Visual Molecular Dynamics. *J. Mol. Graph. Model.* **1996**, *14* (1), 33–38. https://doi.org/10.1016/0263-7855(96)00018-5.

(13) The pandas development team. Pandas-Dev/Pandas: Pandas, 2024. https://doi.org/10.5281/zenodo.10957263.

(14) Harris, C. R.; Millman, K. J.; van der Walt, S. J.; Gommers, R.; Virtanen, P.; Cournapeau, D.; Wieser, E.; Taylor, J.; Berg, S.; Smith, N. J.; Kern, R.; Picus, M.; Hoyer, S.; van Kerkwijk, M. H.; Brett, M.; Haldane, A.; del Río, J. F.; Wiebe, M.; Peterson, P.; Gérard-Marchant, P.; Sheppard, K.; Reddy, T.; Weckesser, W.; Abbasi, H.; Gohlke, C.; Oliphant, T. E. Array Programming with NumPy. *Nature* **2020**, *585* (7825), 357–362. https://doi.org/10.1038/s41586-020-2649-2.

(15) Pedregosa, F.; Varoquaux, G.; Gramfort, A.; Michel, V.; Thirion, B.; Grisel, O.; Blondel, M.; Prettenhofer, P.; Weiss, R.; Dubourg, V.; Vanderplas, J.; Passos, A.; Cournapeau, D.; Brucher, M.; Perrot, M.; Duchesnay, É. Scikit-Learn: Machine Learning in Python. *J. Mach. Learn. Res.* **2011**, *12* (85), 2825–2830.

(16) Seabold, S.; Perktold, J. Statsmodels: Econometric and Statistical Modeling with Python. In *Proceedings of the 9th Python in Science Conference*; Walt, S. van der, Millman, J., Eds.; 2010; pp 92–96. https://doi.org/10.25080/Majora-92bf1922-011.

(17) Virtanen, P.; Gommers, R.; Oliphant, T. E.; Haberland, M.; Reddy, T.; Cournapeau, D.; Burovski, E.; Peterson, P.; Weckesser, W.; Bright, J.; van der Walt, S. J.; Brett, M.; Wilson, J.; Millman, K. J.; Mayorov, N.; Nelson, A. R. J.; Jones, E.; Kern, R.; Larson, E.; Carey, C. J.; Polat, İ.; Feng, Y.; Moore, E. W.; VanderPlas, J.; Laxalde, D.; Perktold, J.; Cimrman, R.; Henriksen, I.; Quintero, E. A.; Harris, C. R.; Archibald, A. M.; Ribeiro, A. H.; Pedregosa, F.; van Mulbregt, P. SciPy 1.0: Fundamental Algorithms for Scientific Computing in Python. *Nat. Methods* **2020**, *17* (3), 261–272. https://doi.org/10.1038/s41592-019-0686-2.

(18) Michaud-Agrawal, N.; Denning, E. J.; Woolf, T. B.; Beckstein, O. MDAnalysis: A Toolkit for the Analysis of Molecular Dynamics Simulations. *J. Comput. Chem.* **2011**, *32* (10), 2319–2327. https://doi.org/10.1002/jcc.21787.

(19) Gowers, R. J.; Linke, M.; Barnoud, J.; Reddy, T. J. E.; Melo, M. N.; Seyler, S. L.; Domański, J.; Dotson, D. L.; Buchoux, S.; Kenney, I. M.; Beckstein, O. MDAnalysis: A Python Package for the Rapid Analysis of Molecular Dynamics Simulations. *Proceedings of the 15th Python in Science Conference* **2016**, 98–105. https://doi.org/10.25080/Majora-629e541a-00e.

(20) Scikit-Learn-Contrib/Skope-Rules, 2024. https://github.com/scikit-learn-contrib/skope-rules (accessed 2024-04-15).

(21) Scherer, M. K.; Trendelkamp-Schroer, B.; Paul, F.; Pérez-Hernández, G.; Hoffmann, M.; Plattner, N.; Wehmeyer, C.; Prinz, J.-H.; Noé, F. PyEMMA 2: A Software Package for Estimation, Validation, and Analysis of Markov Models. *J. Chem. Theory Comput.* **2015**, *11* (11), 5525–5542. https://doi.org/10.1021/acs.jctc.5b00743.

(22) Parr, T. Parrt/Dtreeviz, 2024. https://github.com/parrt/dtreeviz (accessed 2024-04-15).

(23) Waskom, M. L. Seaborn: Statistical Data Visualization. *J. Open Source Softw.* **2021**, *6* (60), 3021. https://doi.org/10.21105/joss.03021.

(24) Hunter, J. D. Matplotlib: A 2D Graphics Environment. *Comput. Sci. Eng.* **2007**, *9* (3), 90–95. https://doi.org/10.1109/MCSE.2007.55.
